# Identification of Crohn’s disease-associated molecular signatures by integrative bioinformatics analysis and experimental validation

**DOI:** 10.3389/fimmu.2026.1955481

**Published:** 2026-09-29

**Authors:** Peihong Li, Siqing Guo, Hongyi Hu, Linda Zhong, Changqin Liu, Kexin Zeng, Jiang Lin, Boyun Sun

**Affiliations:** 1 Department of Gastroenterology, Longhua Hospital, Shanghai University of Traditional Chinese Medicine, Shanghai, China; 2 Department of Coloproctology, Shanghai Tenth People’s Hospital, Shanghai, China; 3 School of Biological Sciences, Nanyang Technological University, Singapore, Singapore; 4 Department of Gastroenterology, Shanghai Tenth People’s Hospital, Shanghai, China; 5 College of Plant Protection, Hunan Agricultural University, Changsha, Hunan, China

**Keywords:** bioinformatics analysis, biomarker, crohn’s disease, immune infiltration, machine learning, mendelian randomization

## Abstract

**Background:**

Crohn’s disease (CD) is a chronic inflammatory bowel disease with rising global incidence. Its multifactorial etiology involves genetic, environmental, microbial, and immunological factors. Current diagnostic methods are invasive and costly, underscoring the need for improved biomarkers and non-invasive tools for early detection and monitoring.

**Methods:**

Using bioinformatics and machine learning, we analyzed datasets from the Gene Expression Omnibus (GEO) to identify diagnostic biomarkers and immune infiltration patterns. Multi-omics integration and immune profiling were employed. Differential gene expression, Weighted Gene Co-expression Network Analysis (WGCNA), and Mendelian randomization (MR) identified causal genes. Machine learning (12 algorithms, 113 combinations) developed diagnostic models, validated via Receiver Operating Characteristic (ROC) analysis. Protein-protein interaction network, immune infiltration, and gene enrichment analysis identified biological pathways or functions of key genes. Single-cell sequencing validated key findings. Key genes were experimentally validated through immunohistochemistry (IHC), quantitative polymerase chain reaction (qPCR) and Western blot (WB).

**Results:**

A total of 502 differentially expressed genes (DEGs) were identified, with WGCNA highlighting a key module enriched in immune pathways (IL-17, NF-κB). Machine learning selected 96 genes, and MR identified Fc gamma receptor IIa (FCGR2A) (OR = 1.38, p=0.043) and Serpin family B member 9 (SERPINB9) (OR = 1.27, p=0.001) as candidate biomarkers with evidence supporting potential causal associations with CD. FCGR2A and SERPINB9 were identified as candidate diagnostic biomarkers with Area Under the Curves (AUCs) of 0.788(95% CI, 0.722-0.853) and 0.789(95% CI, 0.707-0.871) and correlated with neutrophil and M1 macrophage infiltration. Single-cell analysis revealed cell-specific expression: FCGR2A in neutrophils/monocytes and SERPINB9 in B cells. IHC, qPCR and WB confirmed their upregulation in CD tissues. A nomogram integrating both genes showed promising predictive performance (C-index=0.819) and net benefit in decision curve analysis.

**Conclusion:**

FCGR2A and SERPINB9 represent candidate molecular biomarkers associated with CD, although FCGR2A largely reflects neutrophil-associated inflammation. The two-gene model showed preliminary discriminatory performance, but independent prospective validation and comparison with established clinical biomarkers are required before clinical application.

## 1 Background

Crohn’s disease (CD) is a chronic, relapsing inflammatory bowel disease (IBD) that can affect any part of the gastrointestinal tract, but most commonly involves the terminal ileum and colon. Characterized by transmural inflammation, CD often presents with symptoms such as abdominal pain, diarrhea, weight loss, and fatigue, leading to significant impairments in quality of life and a heightened risk of complications such as strictures, fistulas, and abscesses (1). The etiology of CD is multifactorial, involving genetic susceptibility, environmental factors, microbial dysbiosis, and immune system dysregulation, but its exact pathogenesis remains incompletely understood (2).

The growing global prevalence of CD poses significant challenges for healthcare systems, with rising incidence rates particularly in developed countries (3). Despite the availability of various treatment options, including immunosuppressants and biologics, many patients experience refractory disease or relapse, underscoring the need for early diagnosis, effective treatment stratification, and better disease monitoring. Currently, the diagnosis of CD relies on a combination of clinical, endoscopic, histological, and radiological findings, which may be invasive and costly, limiting their widespread use for regular monitoring or early detection. Therefore, there is a need for molecular biomarkers that could complement existing diagnostic approaches and facilitate the identification of patients who may warrant further clinical evaluation.

Biomarkers represent a promising avenue for addressing these challenges, offering the potential for non-invasive, accurate, and dynamic tools to aid in diagnosis, predict disease course, and assess treatment responses (4, 5). However, due to the heterogeneity of CD and the complex interplay of its pathogenic mechanisms, the identification of reliable biomarkers has proven difficult. In particular, understanding the immune landscape of CD is crucial, as immune dysregulation is a central feature of its pathology. Exploring immune cell infiltration and immune-related biomarkers can provide valuable insights into the molecular mechanisms driving disease progression and identify new therapeutic targets.

Advances in bioinformatics and machine learning have enabled researchers to harness high-throughput omics data, such as transcriptomics, proteomics, and metabolomics, to discover novel biomarkers and unravel the intricate molecular networks involved in diseases like CD (6–8). These computational approaches allow for the integration of large-scale data from diverse biological sources, facilitating the identification of key genes, pathways, and immune cell populations implicated in disease pathogenesis. Furthermore, machine learning models can be applied to develop predictive tools that offer high sensitivity and specificity in distinguishing between active and quiescent disease states, as well as in forecasting patient outcomes.

In this study, we aimed to identify and characterize molecular signatures associated with CD and to evaluate their potential utility as complementary biomarkers for CD identification. We integrated transcriptomic data with WGCNA, machine learning, Mendelian randomization (MR), immune infiltration analysis, and single-cell RNA sequencing to identify and characterize candidate genes. FCGR2A and SERPINB9 were subsequently evaluated at the transcriptomic and experimental levels, and their diagnostic discrimination was assessed using ROC analysis. We further constructed a nomogram integrating the two genes to explore their potential value as a complementary molecular prediction tool rather than a replacement for established clinical, endoscopic, histological, or laboratory assessments. Given the observed association of FCGR2A with neutrophilic infiltration, we also examined the relationship between gene expression and immune cell composition to clarify whether its diagnostic signal was independent of immune infiltration. The intended target population of this study is patients undergoing evaluation for CD, particularly those in whom molecular information may complement conventional clinical assessment. The proposed model is intended to support, rather than replace, established diagnostic approaches, and its incremental value over routinely used clinical parameters remains to be determined in future independent clinical cohorts. The detailed analytical workflow is shown in **Figure 1**.

**Figure 1:**
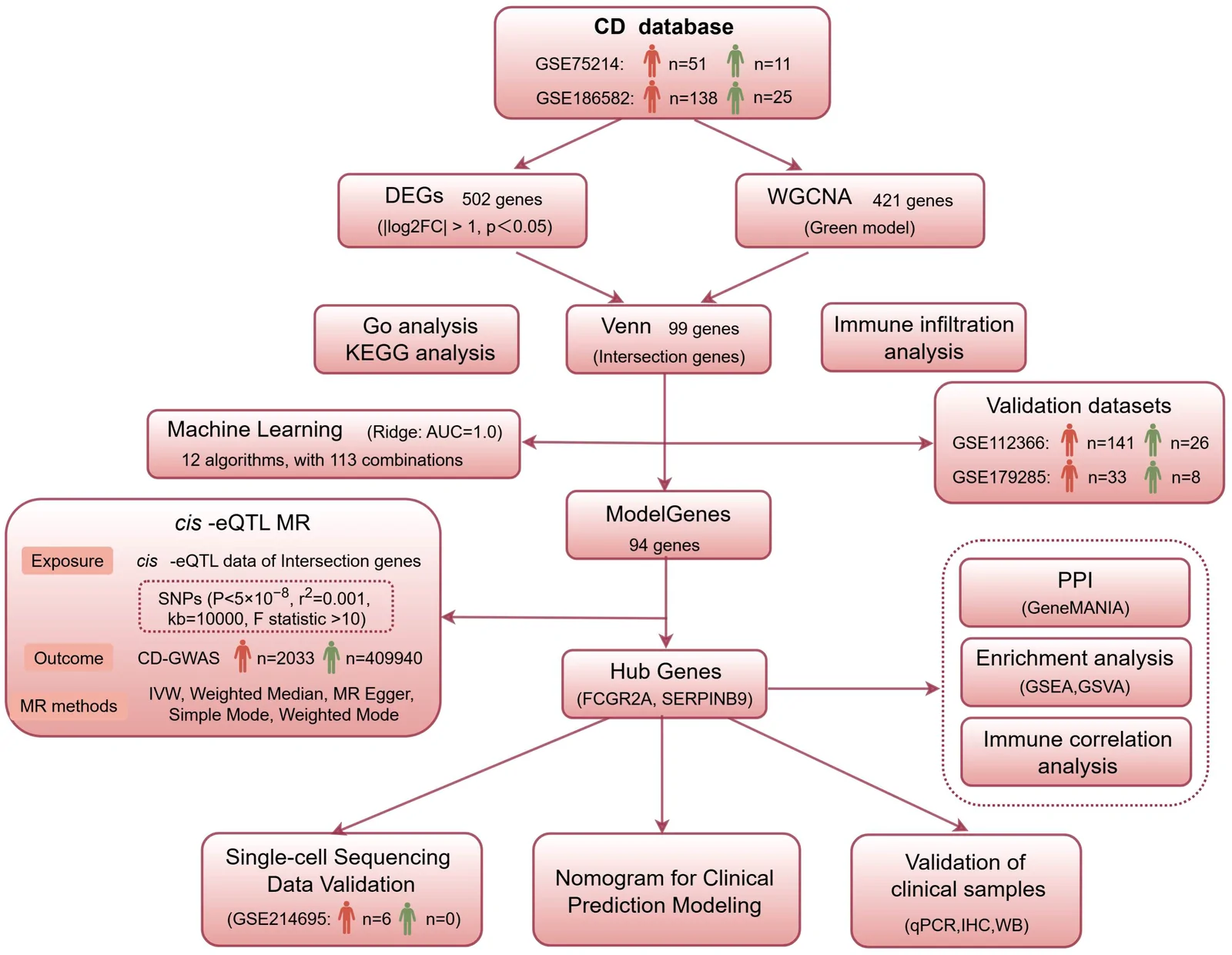
The overall flowchart of this research.

## 2 Materials and methods

### 2.1 Data collection

Data for this study were collected from publicly available databases, including GEO database (9) (https://www.ncbi.nlm.nih.gov/geo/) and the IEU Open GWAS Project(https://gwas.mrcieu.ac.uk/). Microarray datasets from GEO were restricted to ileal samples relevant to the present analysis. For GSE75214, ileal CD and control samples were selected. For GSE186582, ileal samples from CD patients and non-IBD controls were included. For GSE112366, only baseline ileal samples collected before the study treatment were analyzed, whereas post-treatment samples were excluded. For GSE179285, terminal ileal biopsy samples were selected. Treatment history was not uniformly available or comparable across all public datasets and was therefore considered a potential source of cohort heterogeneity. Mendelian randomization (MR) analysis was performed using *cis*-eQTL data from the IEU database and GWAS outcome data from the FinnGen database (K11_CD_STRICT2; n cases = 2033, n controls = 409,940). Single nucleotide polymorphisms (SNPs) were selected as instrumental variables based on genome-wide significance and independence criteria, applying rigorous statistical filters to identify key genes. Single-cell RNA sequencing (scRNA-Seq) data from GEO (GSE214695) were processed through quality control, PCA integration, and cell type annotation to analyze and validate the expression and distribution of key genes in CD intestinal tissues. The included studies were approved by the relevant ethical review committees. The basic information of the datasets is detailed in **Table 1**.

**Table 1:** Basic information of datasets used in this study.

Dataset	Type	Platform	Sample	Biopsy tissue source
GSE75214	Microarrays	GPL6244	CD: 51 cases (Active)/ Control: 11 cases	Ileum tissue
GSE186582	Microarrays	GPL570	CD: 138 cases (Active)/ Control: 25 cases	Ileum tissue
GSE112366	Microarrays	GPL13158	CD: 141 cases/ Control: 26 cases	Ileum tissue
GSE179285	Microarrays	GPL6480	CD: 33 cases (Active)/ Control: 8 cases	Ileum tissue
K11_CD_STRICT2	FinnGen	/	CD: 2033 cases/ Control: 409940 cases	Ileum and colon tissue
GSE214695	ScRNA-Seq	GPL18573	CD: 6 cases (Active)/ Control: none	colon tissue

### 2.2 GEO data preprocessing and differentially expressed genes analysis

The GSE75214 and GSE186582 datasets were used for DEGs analysis to investigate molecular differences between the CD and control groups. Expression matrices were normalized using the Normalize Between Arrays function in the “limma” package to ensure consistency across samples. Outlier detection was conducted via hierarchical clustering based on Pearson correlation coefficients, supplemented by Z-score analysis. Samples with a Z-score deviation greater than 3 (|Z| > 3) were considered outliers and excluded from further analysis to prevent potential bias. The merged normalized dataset was batch-corrected using the ComBat function from the “sva” package to reduce inter-batch variability and minimize confounding factors (10). Principal component analysis (PCA) visualization using “ggplot2” and “ggpubr” from the R package (version 4.3.1) to assess the distribution of samples. DEGs were identified using the “limma” package, with thresholds of adjusted p-value< 0.05 and |log2FC| ≥ 1 to ensure statistical and biological significance. A volcano plot was generated to visualize the DEGs, and heatmaps were created using the “heatmap” package to display the expression patterns between the CD and control groups.

### 2.3 Weighted gene co-expression network analysis

We used the WGCNA package in R to construct gene co-expression networks and identify modules of genes with similar expression patterns (11, 12). These modules facilitate the exploration of potential regulatory relationships and help identify gene networks that may be involved in CD. We analyzed two datasets (GSE75214 and GSE186582) and filtered genes by removing those with very low variability across samples (standard deviation< 0.5) to focus on biologically relevant signals. To ensure data quality, we applied hierarchical clustering to remove potential outlier samples. The gene network was constructed by calculating the optimal soft-thresholding power (β=9), which helps determine meaningful connections between genes. We then converted the data into an adjacency matrix and further refined it into a Topological Overlap Matrix (TOM), which measures how closely genes are connected. Gene modules were identified using dynamic tree cutting, with a merging threshold of 0.25 and a minimum of 60 genes per module. For each module, we measured Module Membership (MM), which shows how strongly a gene belongs to a module, and Gene Significance (GS), which indicates how closely a gene is linked to CD. The module most associated with CD was selected (|MM| > 0.8 and p< 0.05), and genes within it were intersected with DEGs and CD-related genes for further analysis.

### 2.4 Functional enrichment analysis

To elucidate the biological significance of the key intersecting genes, functional enrichment analysis was performed using Gene Ontology (GO) and Kyoto Encyclopedia of Genes and Genomes (KEGG) pathway analyses. All analyses were conducted within the R environment utilizing the “clusterProfiler” package. For GO analysis, genes were categorized into three functional domains: Biological Process (BP), Cellular Component (CC), and Molecular Function (MF). The intersecting genes were first converted to Entrez IDs using the “org.Hs.eg.db” database. Enrichment analysis was performed with a significance threshold set at p< 0.05 and an adjusted p-value< 0.05. KEGG pathway analysis was conducted to identify significantly enriched pathways, using the “enrichKEGG” function with the human genome (organism = “hsa”) as the reference. Multiple testing correction was applied using the false discovery rate (FDR) method, with a threshold of adjusted p< 0.05. The top 30 significantly enriched pathways were visualized using bar plots and dot plots. For data visualization, the “ggplot2,” “circlize,” and “enrichplot” packages were employed. The methodological framework adhered to established protocols (13–15).

### 2.5 Machine learning for candidate diagnostic biomarkers

Machine learning methods were used to build diagnostic models using 12 algorithms, including Lasso, Ridge, Random Forest, Stepglm, XGBoost, Generalized Boosting Regression Modelling, Naive Bayes, Elastic Net, Partial Least Squares Regression for Generalized Linear Models, Linear Discriminant Analysis, glmBoost, and Support Vector Machines, applied individually and in 113 combinations (16). Training datasets (GSE75214 and GSE186582) were preprocessed via normalization, merging, and batch effect correction. Feature selection involved two steps: (1) Algorithm-specific internal validation or cross-validation procedures were applied where applicable. For example, 10-fold cross-validation was used for regularized regression models, and (2) excluding models with fewer than five selected features. Gene selection focused on intersecting genes across datasets, refined using a reference list of intersecting genes from DEGs and WGCNA results. Randomization with a fixed seed ensured reproducibility. Candidate models were evaluated using the area under the curve (AUC) in the training datasets and external validation cohorts. The AUC values across these cohorts were used for comparative evaluation of candidate models, and the model with the highest overall mean AUC was selected as the final model. The external validation cohorts were not used for model fitting or feature selection; however, their AUC values contributed to the comparative evaluation and final model selection. Therefore, these cohorts were considered external validation cohorts rather than completely independent, untouched test sets. Outputs included classification results, risk scores, selected genes, and AUC visualizations, providing a robust framework for diagnostic modelling (17). To further evaluate the clinical utility of Fc gamma receptor IIa (*FCGR2A)* and Serpin family B member 9 (*SERPINB9)*, we calculated the positive predictive value (PPV) and negative predictive value (NPV) at the optimal diagnostic thresholds identified using Youden’s Index. The formulas used for these calculations are:


$$PPV=\frac{Se\times Prev}{Se\times Prev+\left(1-Sp\right)\times \left(1-Prev\right)}$$

$$PPV=\frac{Se\times Prev}{Se\times Prev+\left(1-Sp\right)\times \left(1-Prev\right)}$$

where *Se* is sensitivity, *Sp* is specificity, and *Prev* is the prevalence of CD in the analyzed dataset.

### 2.6 MR analysis

To explore whether specific genes identified by machine learning might play a potential causal role in CD, we conducted MR analysis, a method that uses genetic variations as a natural experiment to assess cause-and-effect relationships. We obtained genetic variants called SNPs from the IEU OpenGWAS database that are strongly linked to the expression of candidate genes and used these as instrumental variables (IVs). SNPs were selected based on three criteria: they had to be highly statistically significant (P< 5 × 10^-8^), independent of each other (filtered using a 10,000 kb window and R²< 0.001 based on the 1000 Genomes European reference panel), and strong enough to provide reliable estimates (F-statistic ≥10 to avoid weak instrument bias). The CD outcome data were obtained from the FinnGen database (K11_CD_STRICT2 GWAS dataset), and we applied multiple statistical approaches, including Inverse Variance Weighted (IVW), Weighted Median, MR-Egger, Simple Mode, and Weighted Mode, to ensure that our findings were consistent across different analytical methods. Additionally, we conducted heterogeneity and pleiotropy tests to check whether the genetic instruments were valid and to rule out potential biases in the analysis.

### 2.7 Development and validation of a nomogram-based clinical prediction model

To develop a predictive model for CD, a logistic regression-based nomogram was constructed using the expression levels of key genes as predictive variables. Gene expression data were processed, and a logistic regression model was fitted using the *lrm()* function from the rms package in R. A nomogram was then generated to estimate the probability of CD, with risk probability scales set at 0.0001, 0.1, 0.3, 0.5, 0.7, 0.9, and 0.99. Model performance was assessed using a calibration curve with bootstrap resampling (B = 1000), and the C-index with a 95% confidence interval (CI) was calculated to evaluate model discrimination. To further assess clinical utility, decision curve analysis (DCA) and a clinical impact curve were generated, analyzing the net benefit across different threshold probabilities. All statistical analyses and visualizations were performed using R version 4.3.1 with the rms and rmda packages.

### 2.8 Protein-protein interaction network

we constructed a PPI network for FCGR2A and SERPINB9 using GeneMANIA (http://genemania.org/search/) (18), selecting experimentally validated interactions, co-expression data, and pathway co-membership while excluding predicted and text-mined associations to reduce false positives. The analysis was conducted in Homo sapiens, using the automatic weighting method to integrate multiple data sources, with the top 20 most functionally related genes added to explore potential novel interactors. To ensure the reliability of the network, we cross-referenced key interactions with STRING and BioGRID, performed functional enrichment analysis, and assessed pathway relevance to CD.

### 2.9 Gene set enrichment and variation analysis

Gene Set Enrichment Analysis (GSEA, http://www.broadinstitute.org/gsea) and Gene Set Variation Analysis (GSVA) were applied to investigate pathways and functional changes associated with key genes (19, 20). we stratified CD samples based on the median expression levels of FCGR2A and SERPINB9. Samples with expression levels above the median were categorized as the high-expression group, while those below the median were classified as the low-expression group. GSEA was conducted to identify differentially expressed KEGG and GO pathways between high- and low-expression groups. Gene sets containing 15–500 genes were analyzed using the weighted enrichment method with 1,000 permutations. Significant enrichment was defined as P< 0.05 and a FDR< 0.05. GSVA, a non-parametric and unsupervised method, was employed to quantify pathway-level variations by translating gene-level changes into comprehensive scores, providing insights into functional differences among samples. Gene sets, including “h.all.v7.5.1.symbols”, “c2.cp.kegg.v7.5.1.symbols” and “c2.cp.reactome.v7.5.1.symbols” were retrieved from the Molecular Signatures Database. These scores facilitated the evaluation of potential biological function alterations across different groups.

### 2.10 Immune infiltration analysis and correlation analysis

The CIBERSORT website was used to screen 22 immune cell matrices (21). p-value< 0.05 indicated significant immune cell infiltration. To illustrate the results, the correlation between unique diagnostic markers and infiltrating immune cells was analyzed using the “ggstatsplot” and “ggplot2” packages. A bar plot showed the levels of immune cells in each sample, while a violin plot illustrated the groups with different immune cells. Correlation analysis was then conducted between the key genes and immune cells. To account for immune cell infiltration, we applied Cell Proportion Adjustment (CPA) using a linear regression approach. To ensure the reliability of immune cell composition estimates, we retained only samples with a CIBERSORT deconvolution p-value< 0.05. Immune cell proportions were first estimated with the CIBERSORT algorithm and then used as covariates to adjust gene expression levels. This allowed us to distinguish genes influenced by immune infiltration from those with disease-intrinsic expression changes. A paired t-test was performed to assess significance before and after adjustment, identifying genes whose expression remained independent of immune composition.

### 2.11 Single-cell sequencing data validation

The raw gene expression matrix from the GEO dataset GSE214695 was used in this study, and the data from six active CD patients were integrated using PCA analysis. The raw scRNA-Seq data underwent quality control and normalization. The scRNA-Seq data were used to analyze the expression and distribution of key genes in different cells of CD intestinal tissues. Cells with fewer than 50 detected genes or with a mitochondrial gene proportion >10% were excluded. We extracted the top 1,500 genes with the highest coefficient of variation for further analysis. After quality control, cell clusters were annotated based on marker genes, and the marker genes were determined as the major cell types. The entire process was completed using the Seurat package in R software.

### 2.12 Histological staining

Terminal ileum tissue samples from 6 CD patients and 6 matched healthy controls were used for immunohistochemical analysis at Shanghai Tenth People’s Hospital. The study was approved by the Ethics Committee of Shanghai Tenth People’s Hospital (Approval No.23KT87), and immunohistochemical analysis was performed in accordance with guidelines. Fresh tissue samples were fixed in 4% neutral formaldehyde overnight, dehydrated, embedded in paraffin, and sectioned into 5-μm slices. The sections were dried at 60 °C for 1–2 hours, deparaffinized with xylene, and rehydrated through a graded ethanol series. Antigen retrieval was performed in citrate buffer by microwave heating (high power for 5 min, medium-low power for 15 min), followed by cooling and PBS washes. Endogenous peroxidase activity was blocked with 3% hydrogen peroxide for 15 min. Non-specific binding was blocked with 5% BSA at 37 °C for 15 min. Sections were incubated with primary antibodies—anti-FCGR2A (15625-1-AP, Proteintech, China) and anti-SERPINB9 (67950-1-Ig, Proteintech, China)—at 37 °C for 1–2 hours or at 4 °C overnight, followed by PBS washes. Secondary antibody incubation was performed at 37 °C for 45 min, followed by DAB staining (GK500705, Gene Tech, China) with real-time microscopic monitoring. Hematoxylin counterstaining (30 s-2 min) was followed by differentiation in hydrochloric acid ethanol (3–5 s). The sections were rinsed, dried at 37 °C, and mounted with neutral resin. Images were captured using a Nikon Eclipse C1 fluorescence microscope. The protein expression in the histologic sections was quantized by the immunohistochemistry (IHC) Image Analysis Toolbox plugin in ImageJ 1.54m software (National Institutes of Health, USA), according to the manufacturer’s instructions (https://imagej.nih.gov/ij/plugins/ihc-toolbox/).

### 2.13 Quantitative polymerase chain reaction

Terminal ileum tissue samples from 6 CD patients and 6 matched healthy controls were used for qPCR analysis. Total RNA was extracted from terminal ileal tissue using TRIzol Reagent (Thermo Fisher Scientific, Carlsbad, CA) following the manufacturer’s instructions. The RNA concentration and purity were determined using a UV spectrophotometer (Eppendorf, Germany). Reverse transcription was performed using the Evo M-MLVRT Master Mix (Accurate Biotechnology, cat. no. AG11706, Changsha, China), and RT-qPCR was conducted with the SYBR^®^ Green Pro Taq HS Premix (Accurate Biotechnology, cat. no. AG11740, Changsha, China) according to the manufacturer’s protocol. The RT-qPCR thermocycling conditions were as follows: initial denaturation at 95 °C for 30 s, followed by 40 cycles of denaturation at 95 °C for 5 s and annealing/extension at 60 °C for 30 s. Relative mRNA expression levels were calculated using the 2^–ΔΔCt^ method, with β-actin serving as the internal reference gene. The primer sequences used in this study are listed in **Table 2**.

**Table 2:** The sequence of primers in q-PCR assay.

Gene names	Sequences
FCGR2A	F: GTCAAATGATTGCCATCAGAAAG
	R: ACATGGTCGTTGGGAGGAAG
SERPINB9	F: TGGTTCTTGTCAATGCCATCTAC
	R: AACGTGGCCTCCTGATACATC
β-Actin	F: TGACGTGGACATCCGCAAAG
	R: CTGGAAGGTGGACAGCGAGG

### 2.14 Western blot

Terminal ileum tissue samples from 12 CD patients and 12 matched healthy controls were used for WB analysis. Terminal ileum tissue was homogenized in RIPA buffer (P0013B; Beyotime, Hangzhou, China) with phosphatase inhibitors (S1873; Beyotime), and protein concentration was determined using the bicinchoninic acid assay method. Twenty micrograms of total protein extracted from 100 mg of terminal ileal mucosa was separated on 10% SDS-PAGE and transferred to polyvinylidene difluoride membranes (Millipore). After blocking with 5% skimmed milk in Tris-buffered saline–Tween 20 (TBST), membranes were immunoblotted with primary antibodies against FCGR2A (#60432, Cell Signaling Technology, Shanghai, China), SERPINB9 (#37908, Cell Signaling Technology, Shanghai, China), or GAPDH (#2118, Cell Signaling Technology, Shanghai, China). The primary antibodies were visualized with goat anti-rabbit peroxidase-conjugated antibody (Cell Signaling Technology, Danvers, MA, USA) using an enhanced chemiluminescence detection system (Millipore). The images of blots were acquired by the GBOX Chemi XT4 System (Syngene, Cambridge, UK) and GeneTools software (Syngene) was used for semi-quantitative analysis.

### 2.15 Statistical analysis

All statistical analyses were conducted using R (version 4.3.1), SPSS (version 25.0), and GraphPad Prism (version 10). For differential expression analysis, we applied the limma package for microarray data and DESeq2 for RNA-Seq data, using Benjamini–Hochberg (BH) correction to control the FDR. WGCNA was performed to identify key gene modules associated with CD, and Pearson correlation analysis was used to assess module-trait relationships. Machine learning model performance was evaluated using AUC and 10-fold cross-validation, with Ridge regression selected to minimize overfitting. For the construction of the nomogram-based predictive model, logistic regression was employed using the rms package, with model performance evaluated by the C-index, calibration curve, and DCA. Group comparisons were performed using Wilcoxon rank-sum tests or Student’s t-tests, depending on data distribution (assessed by the Shapiro–Wilk test). Correlation analyses were conducted using Spearman or Pearson correlation coefficients, as appropriate. For qPCR, IHC and WB experiments, results were expressed as means ± standard deviations (SDs), with group comparisons performed using the Student’s t-test. A P-value< 0.05 was considered statistically significant. To improve study transparency and reproducibility, detailed information on clinical sample selection, sample allocation, data preprocessing, statistical analyses, and missing-data handling is provided in **Tables 3**–**5**. **Table 3** summarizes individual-level clinical characteristics and experimental sample allocation, **Table 4** describes the sample selection and exclusion process, and **Table 5** summarizes the preprocessing, statistical procedures, quality control, and missing-data handling.

**Table 3:** Individual-level clinical characteristics and sample allocation.

Patient/control ID	Age	Sex	Tissue	IHC	qPCR	WB	Treatment status	Inclusion criteria	Exclusion criteria	Final analysis
CD01	55	Male	Terminal ileum	Yes	Yes	Yes	Treatment-naive	Yes	None	Yes
CD02	45	Male	Terminal ileum	Yes	Yes	Yes	Treatment-naive	Yes	None	Yes
CD03	33	Male	Terminal ileum	Yes	Yes	Yes	Treatment-naive	Yes	None	Yes
CD04	27	Female	Terminal ileum	Yes	Yes	Yes	Treatment-naive	Yes	None	Yes
CD05	51	Female	Terminal ileum	Yes	Yes	Yes	Treatment-naive	Yes	None	Yes
CD06	24	Female	Terminal ileum	Yes	Yes	Yes	Treatment-naive	Yes	None	Yes
CD07	49	Male	Terminal ileum	No	No	Yes	Treatment-naive	Yes	None	Yes
CD08	56	Female	Terminal ileum	No	No	Yes	Treatment-naive	Yes	None	Yes
CD09	19	Male	Terminal ileum	No	No	Yes	Treatment-naive	Yes	None	Yes
CD10	21	Male	Terminal ileum	No	No	Yes	Treatment-naive	Yes	None	Yes
CD11	53	Female	Terminal ileum	No	No	Yes	Treatment-naive	Yes	None	Yes
CD12	49	Male	Terminal ileum	No	No	Yes	Treatment-naive	Yes	None	Yes
HC01	53	Male	Terminal ileum	Yes	Yes	Yes	/	Yes	None	Yes
HC02	50	Male	Terminal ileum	Yes	Yes	Yes	/	Yes	None	Yes
HC03	61	Male	Terminal ileum	Yes	Yes	Yes	/	Yes	None	Yes
HC04	55	Male	Terminal ileum	Yes	Yes	Yes	/	Yes	None	Yes
HC05	48	Female	Terminal ileum	Yes	Yes	Yes	/	Yes	None	Yes
HC06	30	Female	Terminal ileum	Yes	Yes	Yes	/	Yes	None	Yes
HC07	35	Female	Terminal ileum	No	No	Yes	/	Yes	None	Yes
HC08	45	Male	Terminal ileum	No	No	Yes	/	Yes	None	Yes
HC09	55	Male	Terminal ileum	No	No	Yes	/	Yes	None	Yes
HC10	37	Male	Terminal ileum	No	No	Yes	/	Yes	None	Yes
HC11	47	Male	Terminal ileum	No	No	Yes	/	Yes	None	Yes
HC12	29	Male	Terminal ileum	No	No	Yes	/	Yes	None	Yes

Clinical sample selection. Patients with CD were included if they had a confirmed diagnosis of CD based on an integrated assessment of clinical, laboratory, endoscopic, histological, and radiological findings and met the criteria for active disease, defined as a Crohn’s Disease Activity Index (CDAI) score of >150 and <450. Patients were required to be treatment-naïve at the time of tissue collection and to have adequate terminal ileum tissue samples available for molecular analysis. Healthy controls were individuals without a history of inflammatory bowel disease or other major gastrointestinal disorders who underwent relevant clinical evaluation and had adequate terminal ileum tissue samples available. Participants were excluded if they had other active gastrointestinal inflammatory or infectious diseases, including intestinal tuberculosis, infectious enteritis, ulcerative colitis, ischemic enteritis, or radiation enteritis; severe complications requiring emergency surgical intervention, such as intestinal perforation, intestinal obstruction, or massive gastrointestinal bleeding; pregnancy, planned pregnancy, or breastfeeding; severe systemic diseases requiring pharmacological treatment that could substantially affect inflammatory or immune responses; severe psychiatric or psychological disorders; malignant tumors; previous CD-directed treatment; or inadequate tissue samples for molecular analysis.

**Table 4:** Clinical sample selection and exclusion summary.

Stage	CD, n	Healthy controls, n	Description
Initially assessed	15	17	Patients/samples undergoing terminal ileum tissue collection
Excluded before enrollment	3	5	Did not meet inclusion criteria or met exclusion criteria
Eligible participants	12	12	Met all predefined inclusion criteria and none of the exclusion criteria
IHC analysis	6	6	Adequate tissue and evaluable staining
qPCR analysis	6	6	Adequate tissue and RNA quality
WB analysis	12	12	Adequate tissue and protein quality
Final experimental cohort	12	12	Included in at least one experimental validation

**Table 5:** Data preprocessing, exclusion criteria, statistical procedures, and missing-data handling.

Analysis	Dataset/samples	Preprocessing	Exclusion criteria	Statistical/model procedure	Missing-data handling
DEGs	GSE75214, GSE186582	Normalization; batch correction	Outliers with |Z| > 3	limma; adjusted P < 0.05; |log2FC| ≥ 1	Incomplete profiles excluded
WGCNA	GSE75214, GSE186582	Gene filtering; SD ≥ 0.5	Potential sample outliers	WGCNA; β=9; |MM| > 0.8; P < 0.05	Incomplete samples excluded
Machine learning	Training datasets	Normalization; batch correction	Insufficient data	12 algorithms; 113 combinations; 10-fold CV	Missing predictors excluded
MR	IEU/FinnGen	SNP filtering; LD clumping	Weak/invalid/dependent SNPs	IVW, MR-Egger, weighted median, simple/weighted mode	SNPs failing QC excluded
Immune infiltration	Training cohort	CIBERSORT deconvolution	CIBERSORT P ≥ 0.05	Correlation + CPA	QC-failing samples excluded
scRNA-seq	GSE214695	QC; normalization; PCA	<50 genes/cell or mitochondrial proportion >10%	Seurat clustering/annotation	Low-quality cells removed; no imputation
IHC	6 CD + 6 HC	Tissue processing/staining	Poor-quality or inadequate sections	Group comparison	Non-evaluable sections excluded
qPCR	6 CD + 6 HC	RNA extraction; RT-qPCR	Poor RNA quality/assay failure	2^-ΔΔCt; group comparison	Failed reactions excluded
WB	12 CD + 12 HC	Protein extraction; SDS-PAGE	Inadequate protein/technical failure	Densitometry; group comparison	Non-evaluable samples excluded

CD, Crohn’s disease; HC, healthy control; IHC, immunohistochemistry; qPCR, quantitative polymerase chain reaction; WB, Western blot; WGCNA, weighted gene co-expression network analysis; MR, Mendelian randomization; CPA, cell proportion adjustment; CV, cross-validation; QC, quality control. Patient IDs and demographic information should be replaced with the actual study records.

## 3 Results

### 3.1 Data processing and identification of DEGs.

We selected the GSE75214 and GSE186582 microarray datasets as the training set, which included a total of 189 samples from patients with CD and 36 samples from healthy controls. Following batch effect correction, these datasets were normalized and merged for subsequent analysis. PCA plots and boxplots before and after batch correction confirmed the successful mitigation of batch effects and the appropriate normalization of the data (**Figures 2A–D**). Using the “limma” package in R software, we performed differential expression analysis on the merged datasets (|log2FC|=1 and adjusted p-value< 0.05), resulting in the identification of 502 DEGs (**Supplementary Table S1**), including 238 upregulated and 264 downregulated genes. The top DEGs were visualized using volcano plots and heatmaps (**Figures 2E–F**).

**Figure 2:**
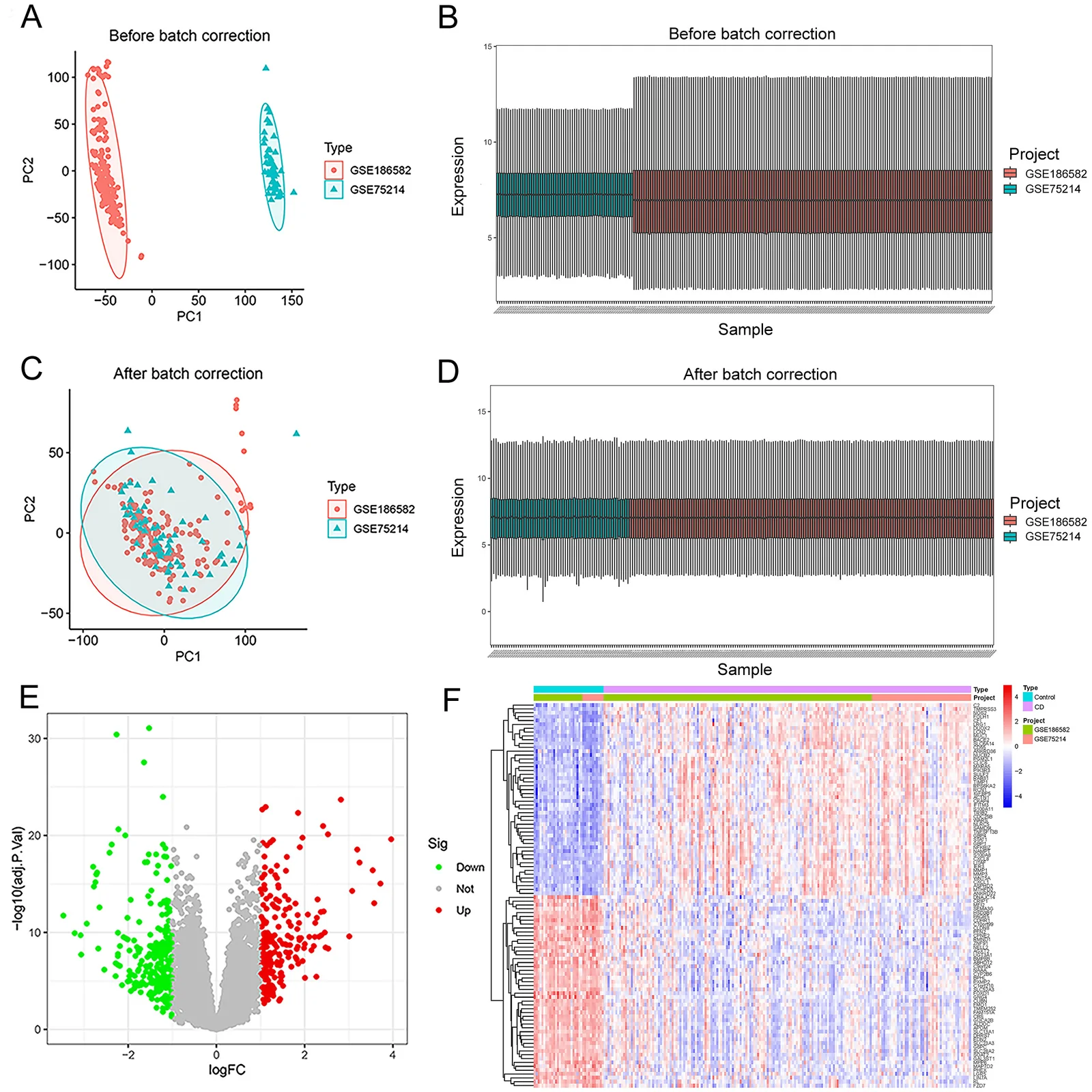
Data processing and screening for DEGs. **(A)** PCA plot before batch correction, showing distinct distributions of the two GEO datasets (GSE75214 and GSE186582, combined n=225: 189 CD, 36 controls), indicating the presence of batch effects due to different experimental samples. The x-axis (PC1) and y-axis (PC2) represent the first and second principal components, respectively, which capture the largest variance in gene expression data. Ellipses represent the 95% confidence intervals for each dataset. **(B)** Box plot before batch correction, where the x-axis represents different samples and the y-axis represents gene expression levels. Large expression differences between experimental samples indicate batch effects. **(C)** PCA plot after batch correction, with the samples randomly reassigned after the removal of batch effects. **(D)** Box plot after batch correction, where gene expression levels are aligned across all samples, reflecting successful removal of batch effects. **(E)** Volcano plot of DEGs identified using the limma package with Benjamini-Hochberg adjusted p< 0.05 and |log2FC| ≥ 1; red = upregulated (n=238), blue = downregulated (n=264). **(F)** Heatmap of the top DEGs (by adjusted p-value); rows represent genes, columns represent samples (n=225); color scale indicates z-scored expression.

### 3.2 WGCNA and identification of key modules

We utilized WGCNA to identify key modules associated with CD in our dataset. Initially, we selected the optimal power value for network construction by generating scatter plots that examined the scale-free topology fit index and mean connectivity across different soft-threshold power values (β=9) (**Figure 3A**). Based on these evaluations, we determined the most suitable power value to ensure that the network conformed to scale-free topology principles. Subsequently, we constructed a gene co-expression network by transforming the similarity matrix into a TOM. Using the 1-TOM measure, we generated a hierarchical clustering dendrogram of all genes in the dataset (**Figure 3B**), and identified eight distinct modules with varying expression patterns based on MM and GS associated with clinical features (**Figure 3C**, **Supplementary Table S2**). Among these, the “green” module emerged as the most crucial, exhibiting the strongest correlation with CD (**Figure 3D**). Further analysis revealed that the scatter plot of MM versus gene significance for the green module demonstrated the highest correlation with CD in the merged dataset (**Figure 3E**).

**Figure 3:**
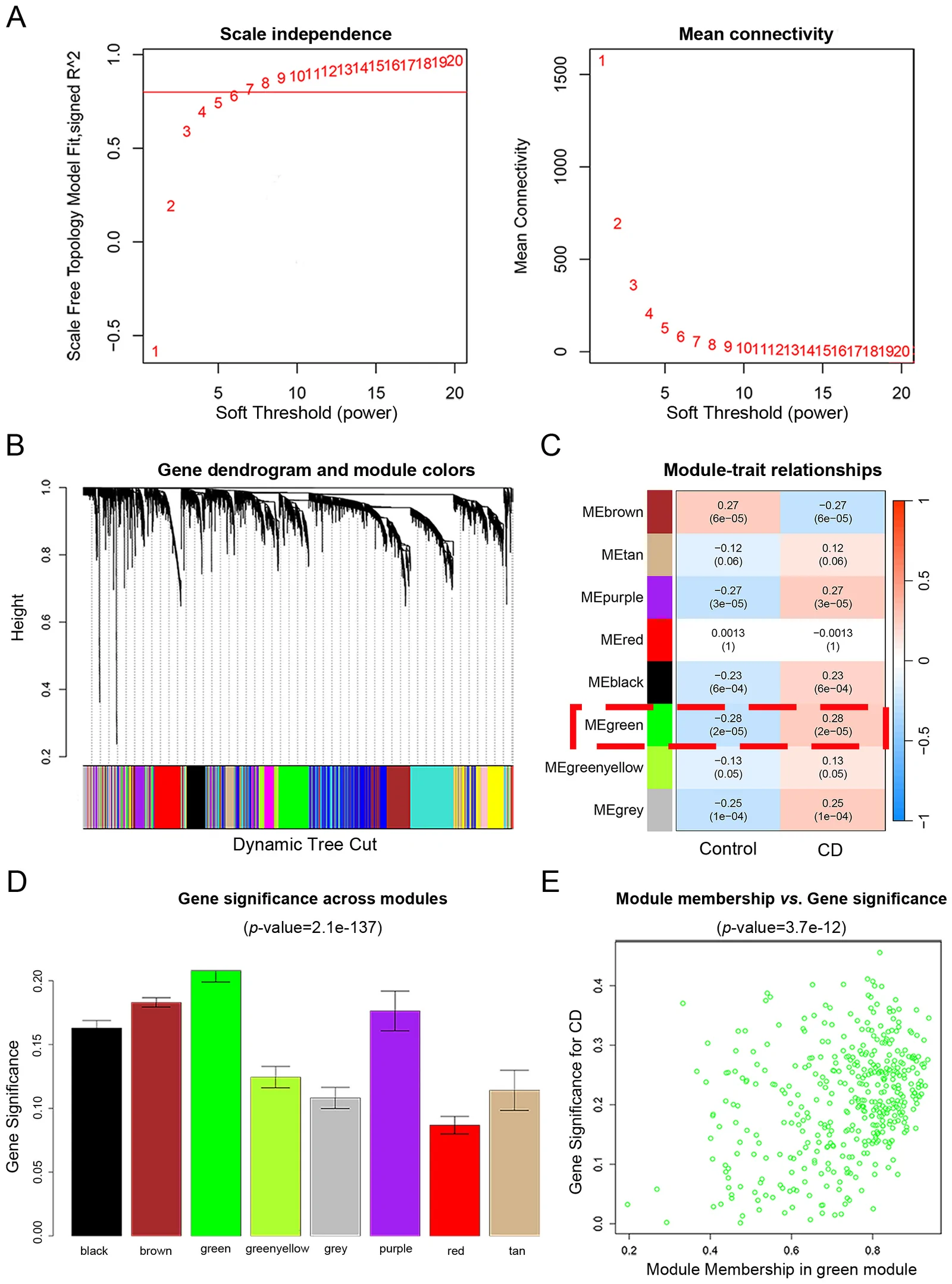
Identification of the GEO merged dataset using WGCNA. **(A)** Scatter plots showing the fit index of module strength classification against power values, average connectivity, and power values. **(B)** Clustering analysis of all genes in the merged dataset based on the Topological Overlap Matrix (1-TOM), where each branch of the clustering tree represents a gene and is reintegrated to construct module genes. **(C)** The significance of genes in each module of the merged dataset is represented by a histogram, with the upper values indicating correlation coefficient size and the lower p-values representing significance, where the “green” module is the most significant. **(D)** A scatter plot merged with a histogram for the “green” module in the merged dataset. **(E)** Scatter plots for modules generated from genes that are positively correlated and maximally correlated with CD in the merged dataset.

### 3.3 Visualization and functional enrichment analysis of intersecting genes

We identified 99 co-expressed genes by Venn diagram analysis that intersected modular genes from WGCNA with DEGs (**Figure 4A**), which may play important roles in the occurrence and progression of CD. Subsequently, we conducted GO and KEGG analyses on these 99 genes to explore their potential functions. The GO analysis described the intersecting genes from three aspects: BP indicating the impact of bacterial-derived molecules (such as lipopolysaccharides), neutrophil migration and chemotaxis, and other related biological processes. CC enrichment covered elements such as cell membranes, granules, and cytoplasmic vesicles. MF were manifested in cytokine activity, chemokine activity, receptor binding activity, G-protein coupled receptor binding, specific immune receptor activity, pattern recognition receptor activity, and complement receptor activity, among others (**Figures 4B, C**). KEGG analysis suggested that the primary pathways involved were the IL-17 signaling pathway, NF-kappa B signaling pathway, Toll-like receptor signaling pathway, TNF signaling pathway, NOD-like receptor signaling pathway, C-type lectin receptor signaling pathway, and AGE-RAGE signaling pathway (**Figures 4D, E**).

**Figure 4:**
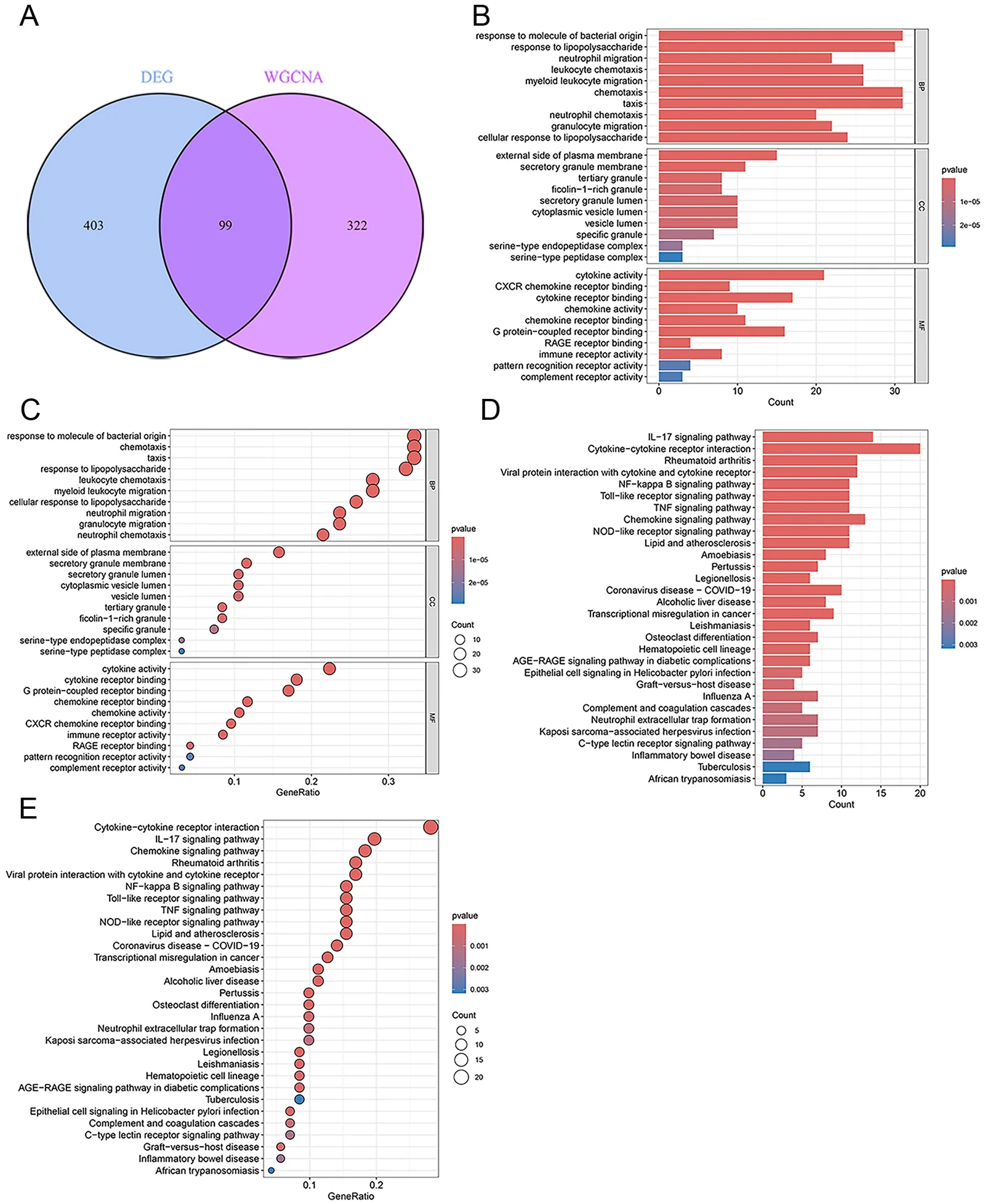
Enrichment Analysis of intersecting genes. **(A)** The Venn diagram illustrates the number of intersecting genes between the DEGs and the “green” module genes identified through WGCNA. **(B)** GO enrichment bar chart for the intersecting genes. The x-axis represents the number of genes associated with each GO term, while the y-axis categorizes terms into the three GO domains: BP, CC, and MF. Color coding corresponds to p-value significance, with darker red indicating higher significance. **(C)** GO enrichment bubble chart, where the x-axis represents the proportion of genes (GeneRatio) associated with each term, and the y-axis categorizes them into BP, CC, and MF. Bubble size corresponds to the number of genes, while color intensity reflects statistical significance (darker red represents lower p-values). **(D)** KEGG enrichment bar chart for the intersecting genes. The x-axis represents the number of genes enriched in each pathway, and the y-axis lists the significantly enriched pathways. Color coding denotes p-value significance, with darker red indicating stronger statistical significance. **(E)** KEGG enrichment bubble chart, with the x-axis representing GeneRatio and the y-axis listing different pathways. Bubble size reflects the number of genes associated with each pathway, while color gradient corresponds to statistical significance (darker red indicates lower p-values).

### 3.4 Identification of diagnostic genes and model construction via machine learning.

To identify diagnostic genes with clinical significance and develop a potential diagnostic model, we integrated 12 different machine learning algorithms in 113 unique combinations to select the most reliable diagnostic model based on 99 co-expressed genes. This analysis was rigorously performed on both the training dataset and two external validation datasets, GSE112366 and GSE179285. We evaluated the overall diagnostic performance of the machine learning models using Receiver Operating Characteristic (ROC) (**Supplementary Table S3**). The Ridge algorithm with the highest average AUC was considered the best model. In the training dataset, the Ridge algorithm shows a perfect ROC curve with an AUC value of 1,000 (95% CI: 1,000-1,000), indicating excellent classification capabilities (**Figures 5A, B**). This performance was further validated in the external validation cohort with an AUC of 0.867 (95% CI: 0.796-0.927) for the GSE112366 dataset (**Figure 5C**) and 0.992 (95% CI: 0.966-1.000) for the GSE179285 dataset (**Figure 5D**). Overall, the model showed comparable discriminatory performance across the evaluated cohorts. 96 key genes were identified by the Ridge algorithm as detailed in **Supplementary Table S4**.

**Figure 5:**
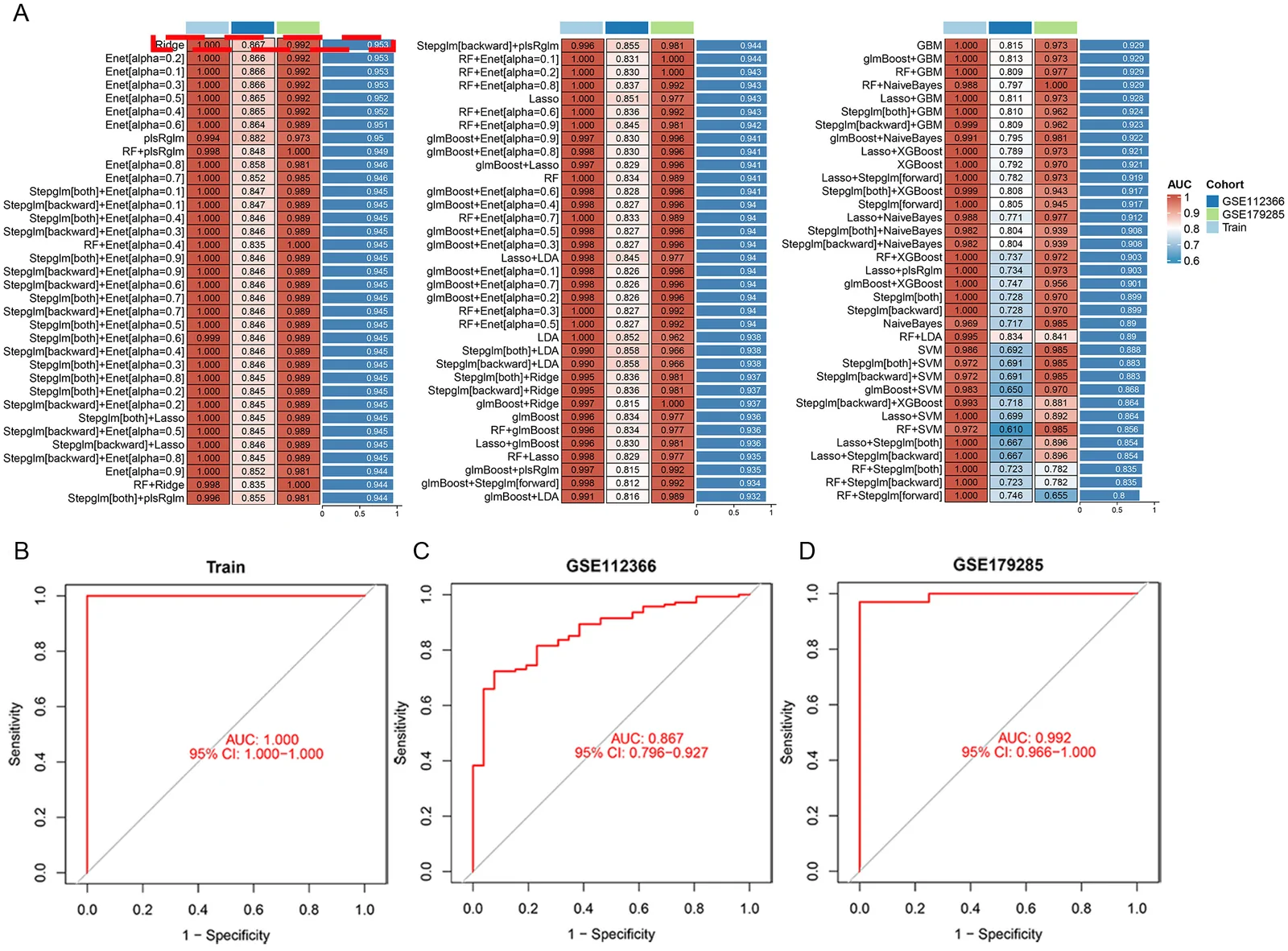
Diagnostic performance of the machine learning models. **(A)** The combinations of 113 machine learning algorithms evaluated through ten-fold cross-validation, with the AUC values representing the diagnostic accuracy of the models across different datasets. The genes selected by the algorithm with the highest average AUC value were used for final model construction. **(B)** ROC curve and AUC value for the merged training set. **(C)** ROC curve and AUC value for the validation set GSE112366. **(D)** ROC curve and AUC value for the validation set GSE179285.

### 3.5 MR analysis of key genes associated with CD

We extracted the *cis*-eQTL data of 96 key genes identified by machine learning-based screening and used them as exposure data, and used CD GWAS data from the FinnGen database as the outcome data. The MR analysis results were primarily determined by the IVW method, excluding horizontal pleiotropy and heterogeneity, and aligned with the differential expression directions observed in the matrix. Finally, two genes were identified as having a causal relationship with CD: FCGR2A and SERPINB9. For FCGR2A, the number of SNPs (nSNP) = 3, IVW: p = 0.043, OR (95% CI) = 1.381 (1.010–1.889); for SERPINB9, nSNP = 3, IVW: p = 0.001, OR (95% CI) = 1.273 (1.101–1.472) (**Figure 6**; **Supplementary Tables S5**–**S7**).

**Figure 6:**
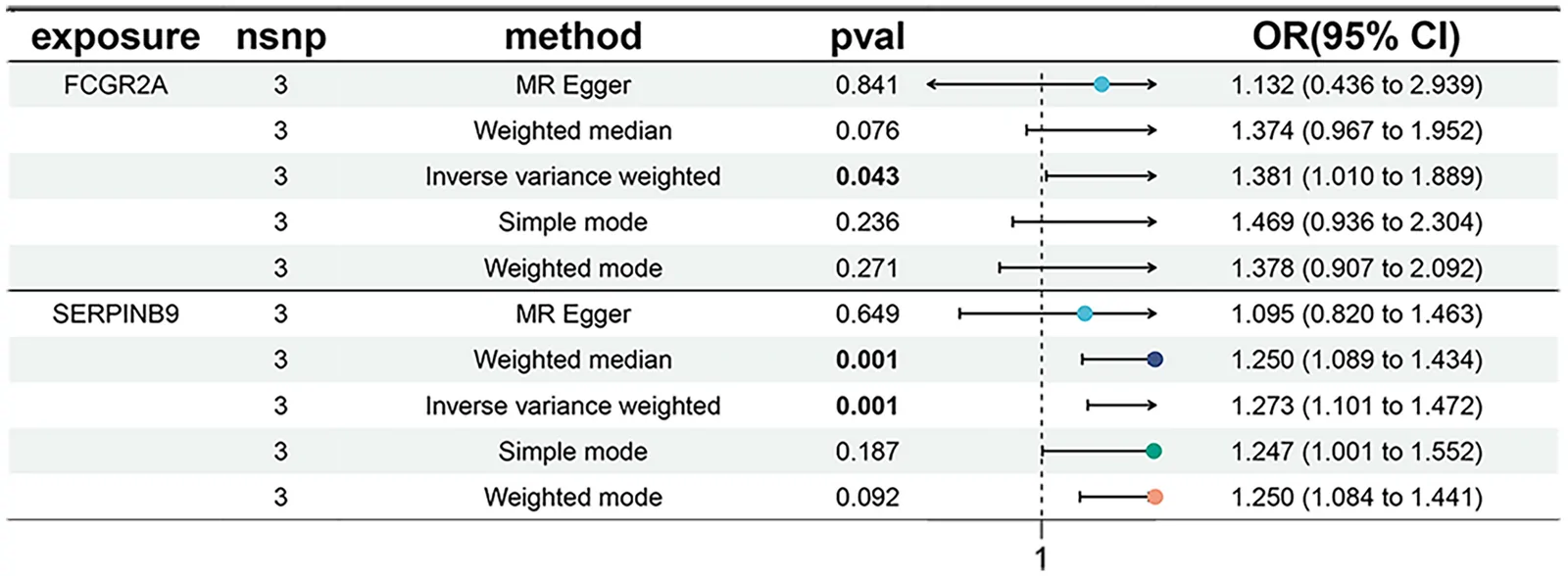
MR analyses of cis-eQTLs. The forest plot illustrates the results of five MR analysis methods, expressed as OR with 95% CI.

### 3.6 Expression analysis and PPI network construction of key genes

We evaluated the expression of the two key genes, FCGR2A and SERPINB9, in the integrated transcriptomic data from the CD training dataset and observed significantly elevated expression in the disease group (**Figure 7A**). To assess the diagnostic utility of these genes, we plotted ROC curves and calculated the AUC. FCGR2A showed an AUC of 0.788 (95% CI, 0.722-0.853), while SERPINB9 showed an AUC of 0.789 (95% CI, 0.707-0.871), indicating moderate discriminatory ability and potential diagnostic value (**Figure 7B**). Furthermore, correlation analysis revealed a statistically significant positive correlation between the two genes (**Figure 7C**). To assess the potential diagnostic value of FCGR2A and SERPINB9, we calculated their PPV and NPV based on Youden’s Index (**Supplementary Table S8**). Both genes showed relatively high PPVs (>86%), suggesting an association between elevated expression and increased likelihood of CD. FCGR2A exhibited high sensitivity (88.65%) but lower specificity (26.92%), indicating a risk of false-positive classification, while SERPINB9 showed a relatively higher specificity (46.15%), although its overall discriminatory performance remained moderate. Although the NPVs were lower (30.4% for FCGR2A and 19.1% for SERPINB9), these findings suggest that the ability of these genes to exclude CD may be limited. Taken together, FCGR2A and SERPINB9 may be considered candidate biomarkers with potential diagnostic value; however, their relatively limited specificity does not support their use as established standalone diagnostic biomarkers. Although we subsequently constructed a two-gene nomogram incorporating FCGR2A and SERPINB9, the present study did not develop or validate a broader multi-marker panel incorporating additional molecular or conventional clinical variables. Therefore, the potential value of these genes within a more comprehensive biomarker panel remains to be investigated. We further explored the potential functional interactions between the proteins encoded by these key genes through a PPI analysis. By utilizing the GeneMANIA database, we constructed an interaction network that highlighted the relationship between the key genes and their associated genes. The analysis revealed that FCGR2A is predominantly involved in immune regulation, including the modulation of cell surface receptor signaling, phagocytosis pathways, and Fc receptor-mediated signaling. Additionally, the associated genes were found to play roles in critical biological processes, such as the regulation of leukocyte degranulation, myeloid cell-mediated immune responses, and immune receptor signaling pathways. The constructed network, including 20 significantly related genes (**Figure 7D**).

**Figure 7:**
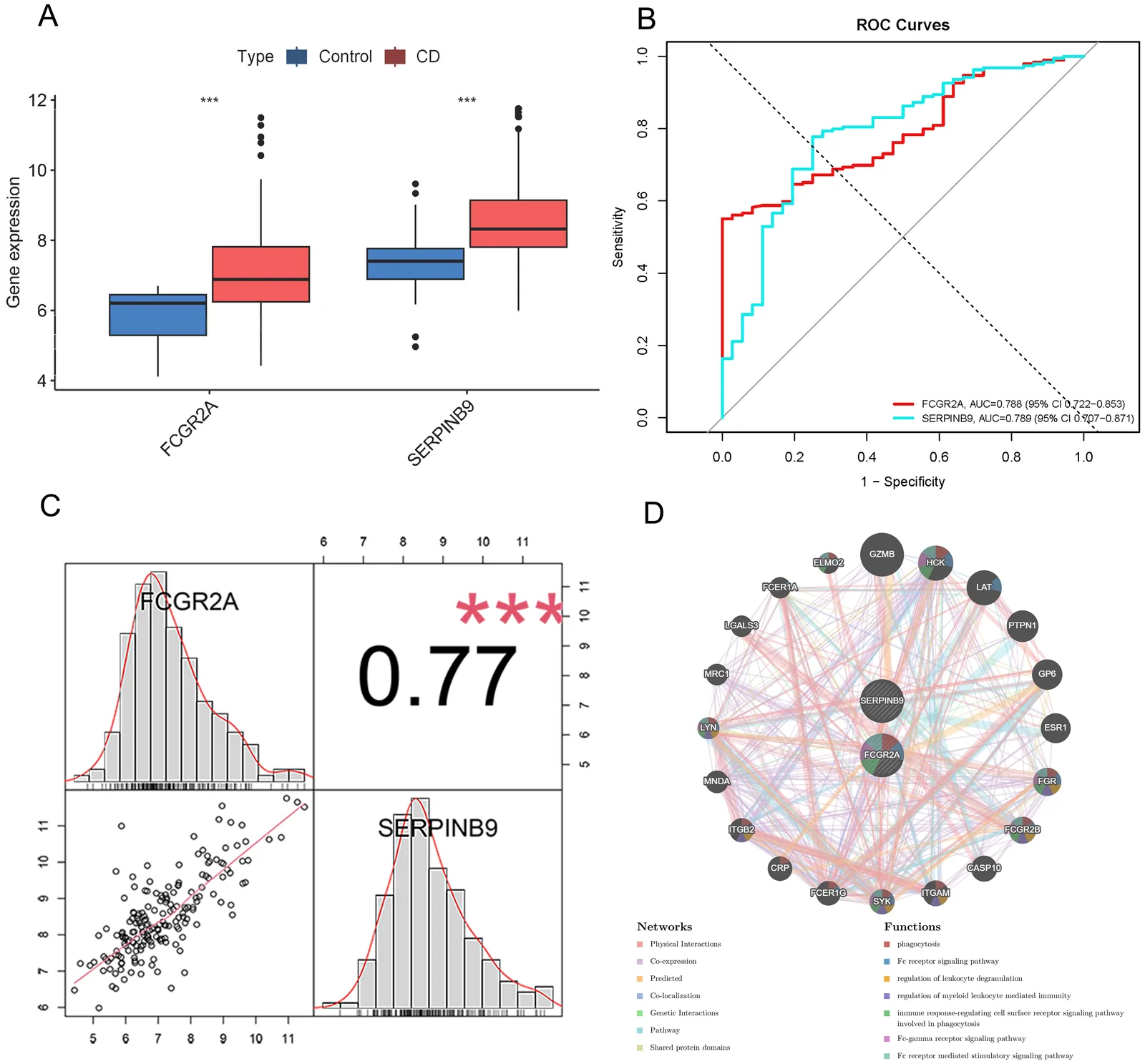
Key gene expression and PPI network analysis. **(A)** The box plots show the expression levels of the key genes in the integrated dataset. The x-axis represents the two key genes, while the y-axis shows their expression levels. Statistical significance between the two groups is indicated by asterisks above the bars, where * represents p< 0.05, ** represents p< 0.01, and *** represents p< 0.001. **(B)** The ROC curves display the diagnostic performance of the key genes, with corresponding AUC values indicating strong discriminatory power between the disease and control groups. **(C)** The correlation matrix plot illustrates the relationship between the two key genes, with the gene names positioned on the diagonal. A correlation coefficient of 0.77 is observed between them, indicating a significant positive correlation (values > 0 indicate positive correlations, while values< 0 indicate negative correlations). Asterisks denote statistically significant differences, where * represents p< 0.05, ** represents p< 0.01, and *** represents p< 0.001. **(D)** The PPI network for the two key genes, constructed using the GeneMANIA database, highlights the interaction between these genes and their associated genes.

### 3.7 Construction and evaluation of a nomogram-based prediction model for CD

To evaluate the predictive value of FCGR2A and SERPINB9 in the diagnosis of CD, we developed a logistic regression-based nomogram as a predictive model. This nomogram integrates the expression levels of the two genes, providing a total score and corresponding disease risk probability for individualized risk assessment (**Figure 8A**). The calibration curve was used to assess the consistency between predicted probabilities and actual incidence rates. The model achieved a C-index of 0.819 (95% CI: 0.769-0.869), indicating good discriminatory ability within the analyzed dataset. The calibration plot demonstrated that the predicted probabilities closely aligned with the observed outcomes, suggesting good model calibration (**Figure 8B**). DCA showed that, across most threshold probability ranges, the predictive model based on FCGR2A + SERPINB9 (red line) provided a higher net benefit compared to both the “no screening” strategy (black line) and the “screen all” approach (gray line), suggesting potential predictive utility within the analyzed dataset (**Figure 8C**). The clinical impact curve further validated the model’s applicability at different high-risk thresholds. The red solid line represents the predicted number of high-risk individuals, while the blue dashed line indicates the number of true-positive cases, demonstrating that the model effectively identifies high-risk individuals while minimizing false positives and false negatives within a reasonable threshold range (**Figure 8D**). However, standardized conventional clinical parameters, such as C-reactive protein (CRP), fecal calprotectin, and endoscopic disease activity measures, were not consistently available across the publicly available datasets included in this study. Therefore, we could not directly compare the FCGR2A + SERPINB9 nomogram with conventional clinical diagnostic parameters or formally determine whether the addition of these two genes provides significant incremental predictive value beyond established clinical indicators. Accordingly, the present nomogram should be considered a candidate prediction model rather than an established clinical diagnostic tool.

**Figure 8:**
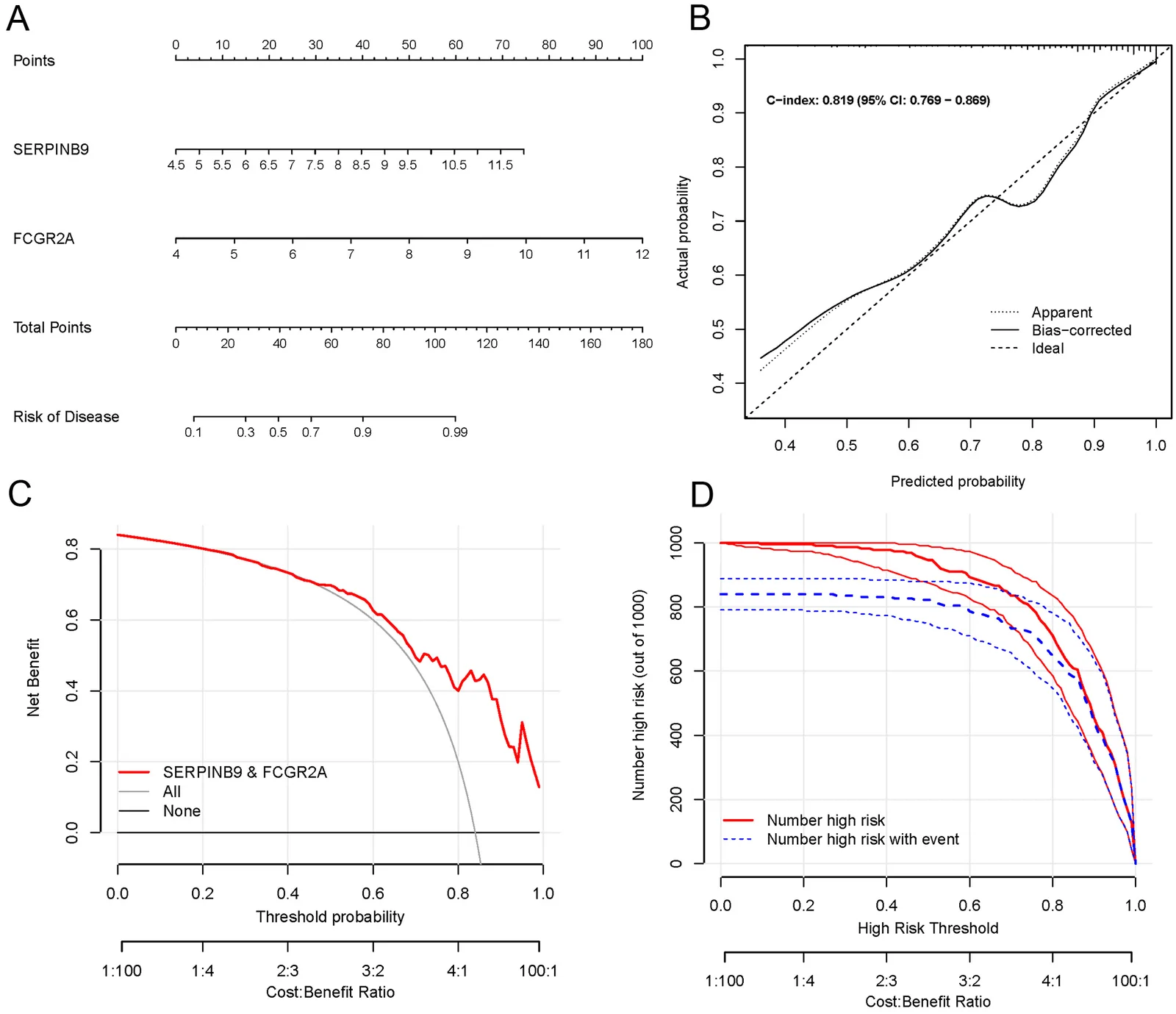
Construction of the clinical prediction model. **(A)** Nomogram for CD risk prediction. The nomogram consists of point scales for SERPINB9 and FCGR2A expression levels, a total points scale, and a corresponding disease risk probability scale. **(B)** Calibration curve. The dotted line represents the apparent calibration, the solid line shows the bias-corrected calibration curve obtained via bootstrap resampling (B = 1000), and the diagonal dashed line indicates the ideal calibration. **(C)** DCA. The red line represents the net benefit of the prediction model across different threshold probabilities. The black line represents no intervention, and the gray line represents treating all individuals. **(D)** Clinical impact curve. The red solid line shows the predicted number of high-risk individuals per 1000 patients at different threshold probabilities, while the blue dashed line represents the number of true-positive cases.

### 3.8 GSEA and GSVA analysis of key genes

In the GSEA analysis, the high-expression groups of FCGR2A and SERPINB9 were predominantly enriched in pathways associated with chemical signaling, cytokine receptor interactions, and Toll-like receptor signaling pathways (**Figures 9A, C**). Conversely, the low-expression groups of FCGR2A and SERPINB9 were primarily enriched in pathways related to drug metabolism (e.g., cytochrome P450), xenobiotic metabolism (e.g., cytochrome P450), and retinol metabolism (**Figures 9B, D**). FCGR2A also demonstrated enrichment in pathways such as drug metabolism involving other enzymes and linoleic acid metabolism, while SERPINB9 showed enrichment in ubiquitin metabolism and steroid biosynthesis processes. Through GSVA analysis, several differentially expressed pathways were identified and presented in the form of a heatmap. The high-expression group of FCGR2A was primarily enriched in pathways including Toll-like receptor signaling, NOD-like receptor signaling, cytokine-cytokine receptor interactions, cytoplasmic DNA sensing, chemokine signaling, and apoptosis-related pathways (**Figure 9E**). The high-expression group of SERPINB9 was mainly enriched in pathways such as chemokine signaling, leukocyte transendothelial migration, cytokine-cytokine receptor interactions, Toll-like receptor signaling, and Fcγ receptor-mediated phagocytosis (**Figure 9F**).

**Figure 9:**
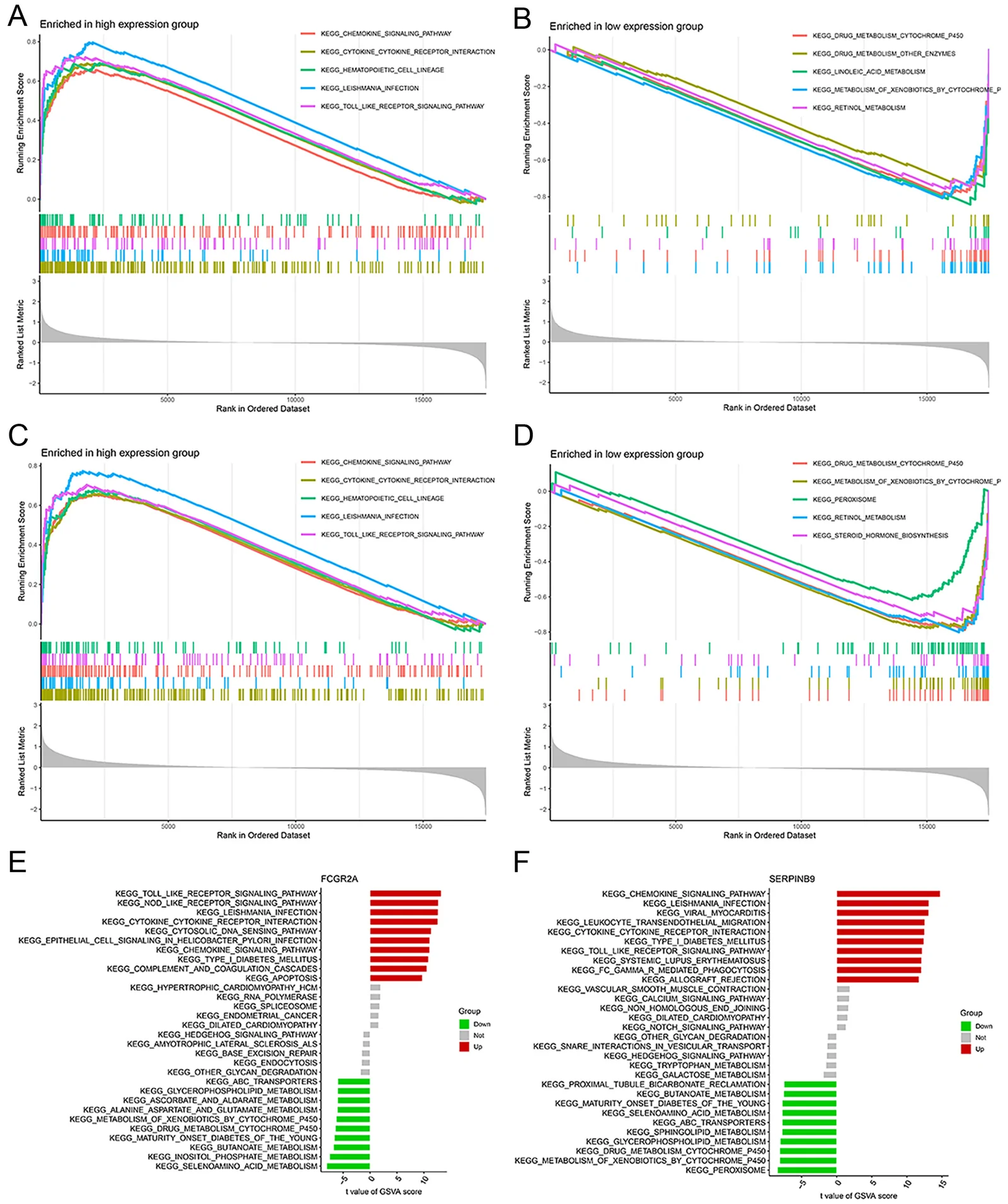
GSEA and GSVA Enrichment Analysis for FCGR2A and SERPINB9. **(A, B)** The top five enriched pathways for both high and low expression groups of FCGR2A. **(C, D)** The top five enriched pathways for both high and low expression groups of SERPINB9. **(E, F)** Differentially enriched pathways for FCGR2A and SERPINB9, with high expression in red and low expression in green.

### 3.9 Immune infiltration and correlation analysis

We conducted an analysis of immune infiltration and visualized the infiltration status of 22 immune cell types in CD samples and healthy control samples within the training set using heatmaps and violin plots. The results from the CIBERSORT algorithm indicated that, compared to normal samples, CD samples generally exhibited higher proportions of Plasma cells, T cells CD4 memory activated, T cells regulatory (Tregs), Monocytes, Macrophages M0, Macrophages M1, Dendritic cells activated, Mast cells activated, and Neutrophils, along with lower proportions of B cells memory, T cells CD8, T cells follicular helper, Macrophages M2, and Mast cells resting (**Figures 10A, B**). Subsequently, we analyzed the correlation between key genes FCGR2A and SERPINB9 and immune cells (**Figure 10C**). To investigate the relationship between gene expression and immune infiltration, we conducted CPA and Spearman correlation analysis. FCGR2A exhibited a strong positive correlation with neutrophils (R = 0.8, P< 2.2e-16) (**Figure 10D**; **Supplementary Figure S1a**), while SERPINB9 showed a strong positive correlation with M1 macrophages (R = 0.64, P< 2.2e-16) and a strong negative correlation with plasma cells (R = -0.61, P< 2.2e-16) (**Figure 10E**; **Supplementary Figure S1b**). CPA analysis further revealed that FCGR2A expression was no longer significant after immune composition adjustment (P = 0.411), suggesting that its upregulation in CD tissues is primarily driven by increased neutrophilic infiltration (**Supplementary Figure S2**; **Supplementary Table S9**). However, despite this dependency on immune cell presence, FCGR2A remains a relevant diagnostic marker, as many established CD biomarkers, such as S100A8/A9 and MPO, are also associated with neutrophil-driven inflammation. In contrast, SERPINB9 remained significantly upregulated after immune adjustment (P = 0.033), indicating that its expression changes are not solely due to immune infiltration but may be linked to the inflammatory microenvironment. These findings suggest that FCGR2A functions as a surrogate marker for neutrophilic inflammation, while SERPINB9 represents a more disease-specific molecular signature. The integration of both markers in a diagnostic model captures immune-driven and intrinsic disease-related expression changes, enhancing the robustness and clinical utility of CD diagnosis.

**Figure 10:**
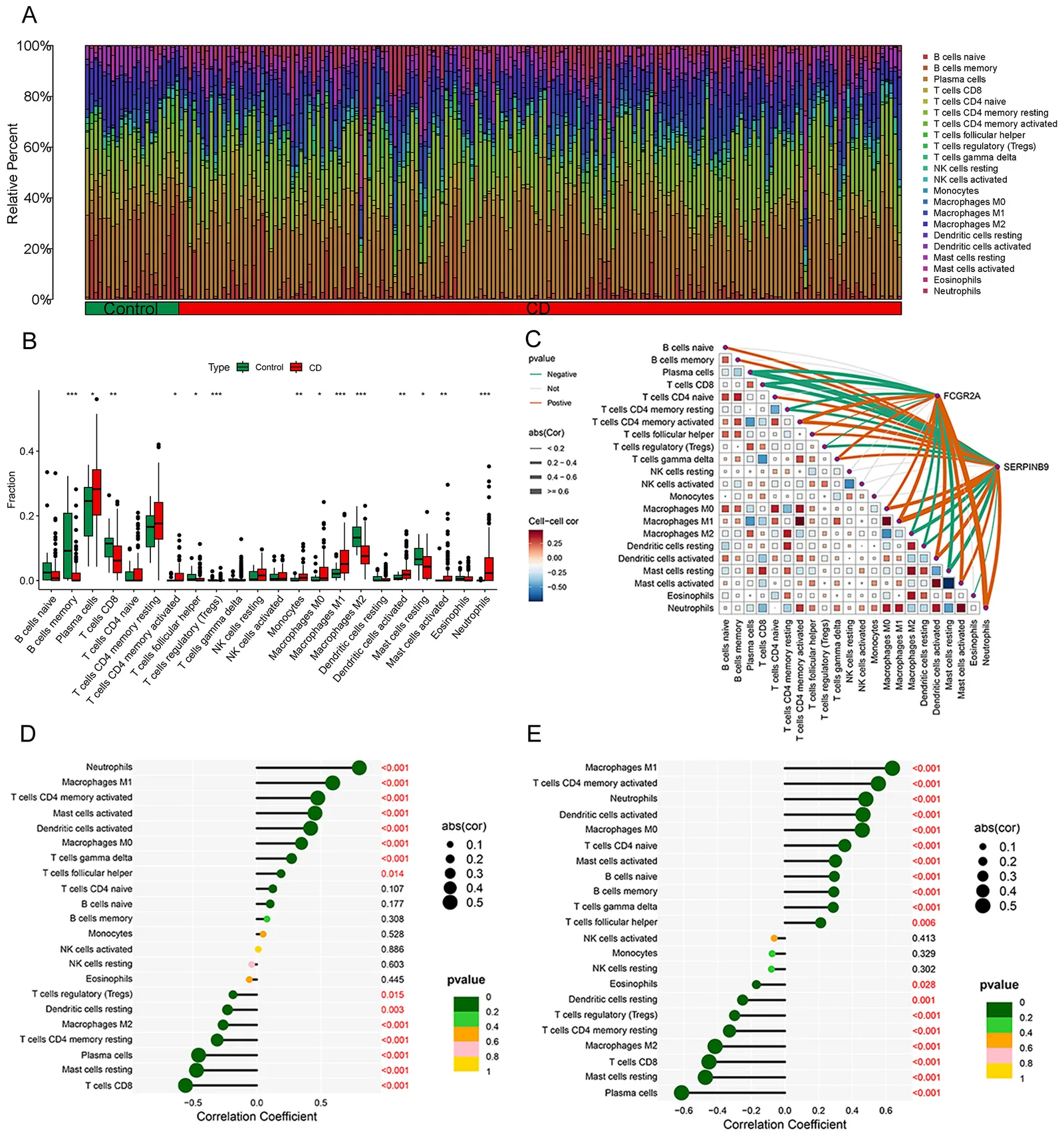
Analysis of immune cell infiltration and its correlation with FCGR2A and SERPINB9. **(A)** Bar graph showing relative immune cell counts in CD patients versus healthy individuals, with different colors for various cell types. **(B)** Violin plots highlight significant differences in immune cell infiltration between groups, denoted by asterisks (*p<0.05, **p<0.01, ***p<0.001). **(C)** Heatmap displays immune cell interactions with color-coded correlations and gene-cell interaction lines indicating positive (yellow) or negative (green) correlations, with line thickness representing correlation strength. **(D, E)** Lollipop plots for FCGR2A and SERPINB9 depict immune cell correlations, with significant associations indicated at p<0.05.

### 3.10 scRNA-seq analysis reveals cell-type-specific expression of key genes

To further investigate the cell type-specific expression patterns of key genes in the colonic tissue of CD patients, this study utilized scRNA-seq data from the GSE214695 database. Cells were clustered into distinct groups based on marker gene expression, and the clustering results were visualized using the t-distributed Stochastic Neighbor Embedding (t-SNE) dimensionality reduction algorithm. A total of seven immune cell subsets were identified, including B cells, neutrophils, monocytes, epithelial cells, T cells, fibroblasts, and natural killer (NK) cells (**Figure 11A**). Further analysis demonstrated that two key genes were expressed across six major cell types, with FCGR2A exhibiting particularly high expression in neutrophils and monocytes. In contrast, SERPINB9 displayed the highest expression levels in B cells (**Figures 11B, C**).

**Figure 11:**
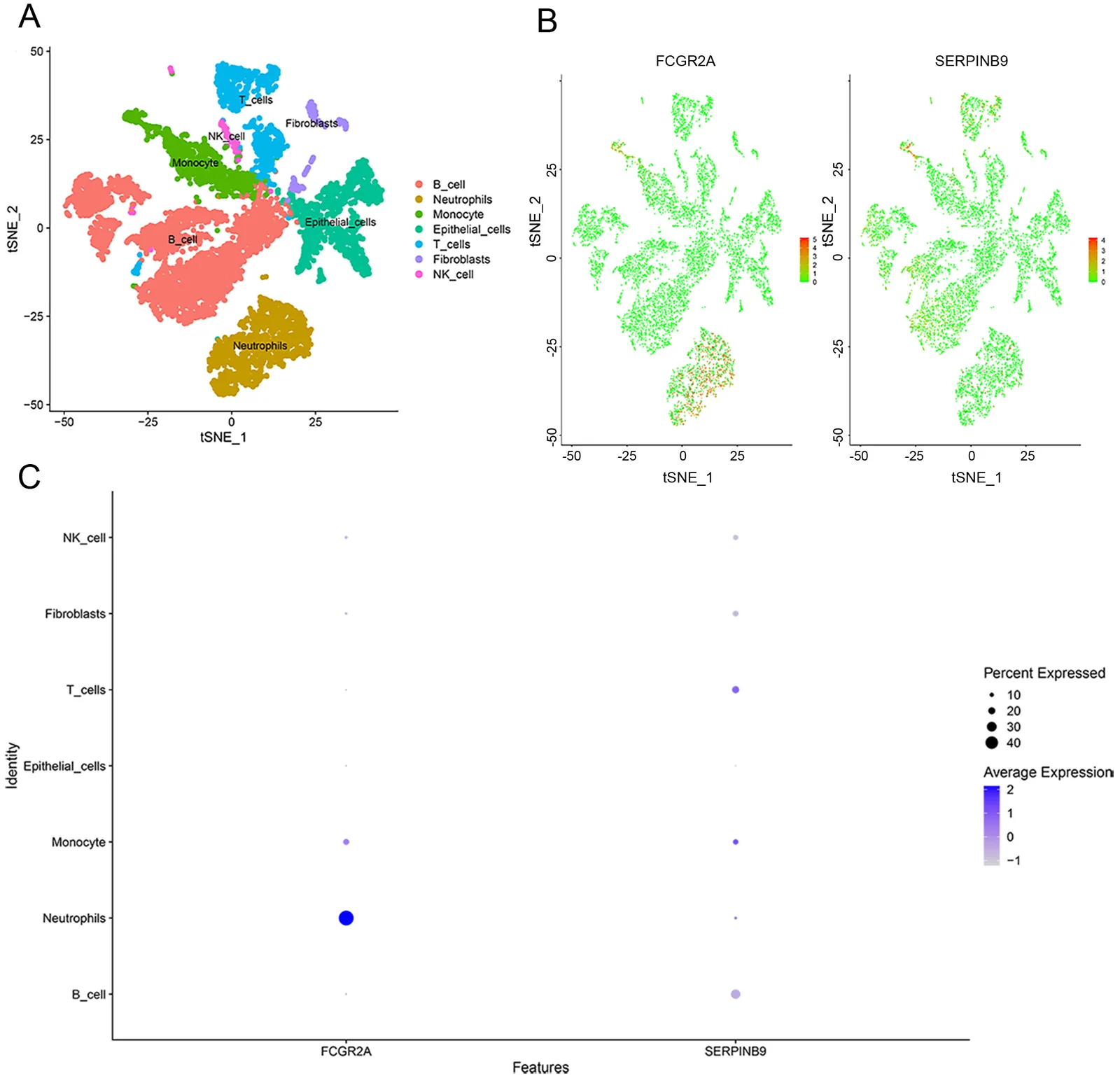
scRNA-Seq analysis of key gene distribution in CD cells. **(A)** Cells were classified into 7 clusters based on cell type-specific marker genes, and visualized using t-SNE dimensionality reduction to show cluster distribution. **(B)** A scatter plot illustrates the distribution and expression of key genes across different cell clusters. **(C)** A bubble plot represents the expression levels of key genes in various cell clusters, with bubble size indicating expression intensity and color reflecting expression level.

### 3.11 Experimental validation of FCGR2A and SERPINB9 upregulation in CD

To validate the bioinformatic findings experimentally, we performed IHC, RT-qPCR, and WB analyses on paired terminal ileum biopsies from CD patients and healthy controls. Immunohistochemical analysis revealed markedly increased positive staining for FCGR2A and SERPINB9 in CD tissues compared to controls (**Figure 12A**). Quantitative assessment of the stained area confirmed a statistically significant increase in SERPINB9 expression (P = 0.0012), and there was a strong increasing trend in FCGR2A expression (P = 0.0456) (**Figure 12B**). RT-qPCR demonstrated significant upregulation of both FCGR2A (P = 0.0002) and SERPINB9 (P = 0.0019) mRNA levels in CD tissues (**Figure 12C**). Similarly, WB analysis confirmed that protein expression levels of FCGR2A and SERPINB9 were significantly higher in the CD group than in the control group (P< 0.001) (**Figures 12D, E**). Since FCGR2A is involved in activating immune cells and SERPINB9 regulates apoptosis and inflammation, their simultaneous upregulation at the mRNA and protein levels strongly suggests their involvement in CD pathogenesis.

**Figure 12:**
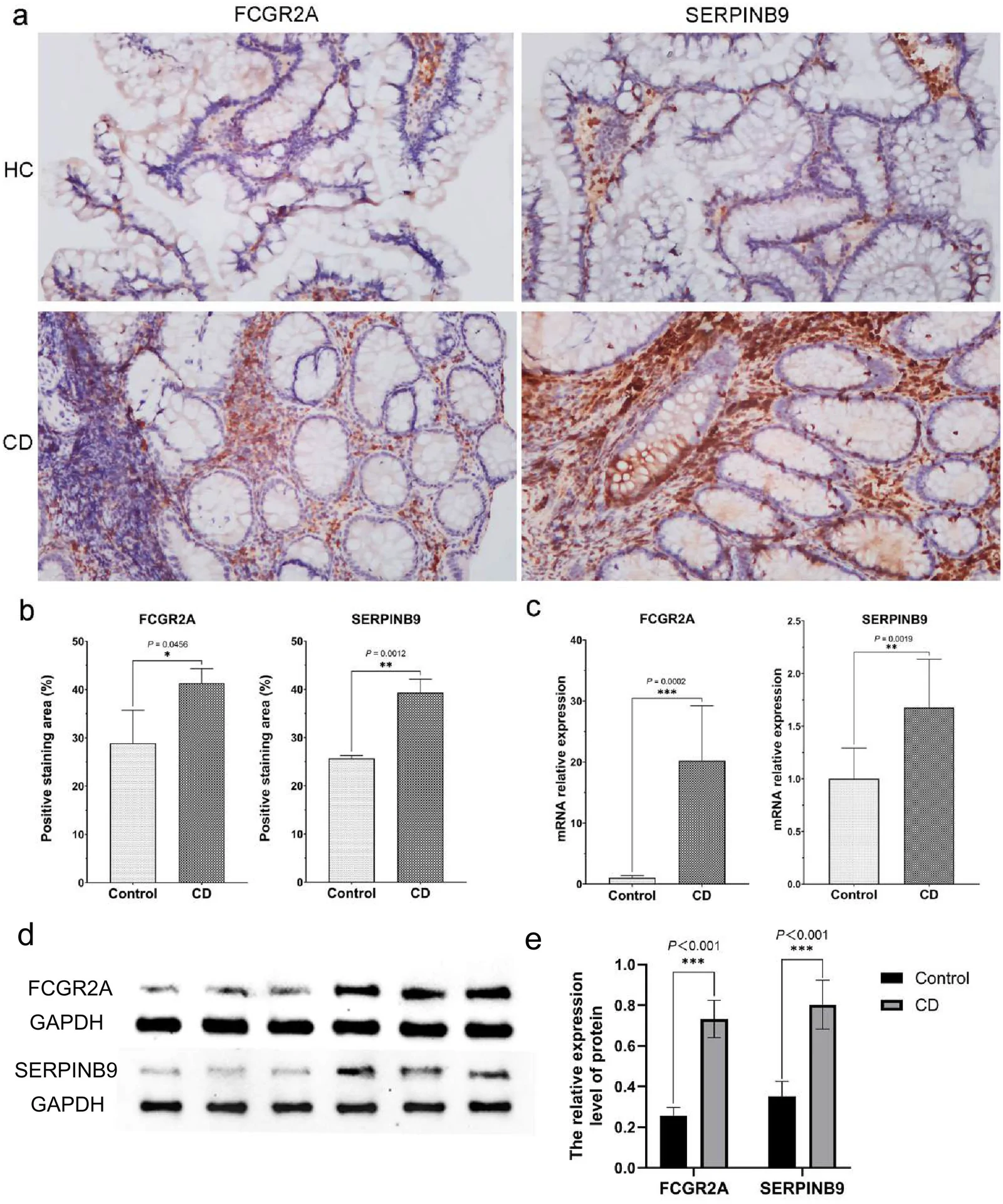
Expression levels of FCGR2A and SERPINB9 were significantly elevated in the CD group. **(A)** Representative IHC images of FCGR2A and SERPINB9 in terminal ileal tissues from CD and healthy control subjects, 200× magnification. **(B)** Analysis of positive staining area percentages in terminal ileal tissues from CD and healthy control subjects (n=6/group). **(C)** Validation of FCGR2A and SERPINB9 mRNA expression by RT-qPCR between CD and healthy control group (n=6/group). **(D, E)** Protein expression of FCGR2A and SERPINB9. GAPDH levels were used as loading controls. Results are expressed as mean ± SD, (n=12/group). *P< 0.05, **P< 0.01, ***P< 0.001.

## 4 Discussion

In this study, we performed a bioinformatics analysis using multi-omics datasets to explore potential biomarkers and immune infiltration patterns associated with CD. By integrating differential gene expression analysis, WGCNA, machine learning, and MR, we identified FCGR2A and SERPINB9 as candidate molecular signatures associated with CD. Additionally, our immune infiltration analysis, supported by validation with single-cell sequencing data, suggests a complex involvement of immune cells in CD, offering potential insights into the disease’s immune landscape. Importantly, the intended target population of this study is patients undergoing clinical evaluation for suspected or established CD, particularly those in whom molecular information may complement conventional clinical assessment. Rather than replacing established diagnostic approaches, the FCGR2A/SERPINB9-based model is intended as a complementary molecular prediction tool to support CD identification and risk assessment. In this context, the model may provide additional molecular information when integrated with existing clinical, endoscopic, histological, and laboratory assessments, although its incremental value over routinely used clinical parameters remains to be determined in independent clinical cohorts.

Our results revealed a total of 502 DEGs that distinguish CD patients from healthy controls, highlighting profound alterations in gene expression associated with the disease. It should be noted that a proportion of these transcriptional changes, as discussed later, may be attributable to shifts in immune cell composition within the inflamed tissue rather than solely cell-intrinsic dysregulation. This finding aligns with previous studies that have reported significant transcriptional changes in CD patients, emphasizing the importance of identifying distinct molecular signatures to improve diagnosis and treatment strategies (6, 7, 22). By employing WGCNA, we identified the “green” module as significantly correlated with CD, underscoring the role of co-expressed gene networks in understanding disease mechanisms (23, 24). Network-based approaches have been instrumental in revealing biological insights that individual gene studies may overlook, providing a holistic view of the molecular landscape of CD. Functional enrichment analysis revealed that the intersecting genes are significantly involved in critical biological processes, including cytokine activity and immune response pathways such as the IL-17 signaling and NF-kappa B pathways. These findings support the central role of immune dysregulation in CD pathogenesis (25–28). The overexpression of cytokines and chemokines may contribute to the chronic inflammation characteristic of CD (29, 30), supporting the notion that targeting these pathways could yield effective therapeutic interventions.

Furthermore, Machine learning results strengthened our findings, allowing for the identification of a potential diagnostic model based on the co-expressed genes. In our study, we leveraged an extensive set of 113 machine learning algorithmic combinations from 12 distinct methods to enhance the diagnostic accuracy of CD, significantly outperforming previous single or limited algorithm approaches (31–35). The Ridge regression model was selected as optimal based on a comprehensive evaluation considering not only its high average AUC across training and validation sets but also its minimal performance gap between them and its inherent L2 regularization, which mitigates overfitting risk in high-dimensional data. The perfect AUC (1.000) observed in the training set was achieved using a 10-fold cross-validation framework, while the model maintained high performance in the external validation cohorts, with AUCs of 0.867 (95% CI: 0.796-0.927) and 0.992 (95% CI: 0.966-1.000). These findings provide preliminary evidence supporting the potential reproducibility of the model across the evaluated cohorts, although further validation in independent prospective cohorts is required to establish its generalizability and clinical utility. This multi-algorithmic strategy highlights the potential value of integrating bioinformatics with machine learning for identifying clinically relevant biomarkers, which could contribute to advancements in personalized medicine and diagnostics for IBDs.

A key outcome of this discovery-phase study is the identification of two key genes, FCGR2A and SERPINB9, which demonstrated a strong association with CD based on integrated analyses. Our MR analysis provided evidence supporting a potential causal link for both genes. These genes are involved in key immune processes, with FCGR2A playing a role in Fcγ receptor-mediated phagocytosis and immune regulation (36), This receptor is crucial for the clearance of immune complexes and the modulation of pro-inflammatory cytokines. In the context of IBD, FCGR2A has been primarily associated with UC, but its potential role in CD remains a subject of investigation. Researchers have identified FCGR2A as a susceptibility locus for IBD via a genome-wide association study, indicating that genetic variations within this gene could affect an individual’s risk of developing IBD (37, 38). This discovery was bolstered by another study which found that the FCGR2A*A519GG genotype was protective against both ulcerative colitis and CD in a Caucasian population (39). This suggests that FCGR2A might contribute to the common genetic susceptibility to both types of IBD. However, the specific mechanisms by which FCGR2A influences susceptibility to CD are not as well established as its role in UC. In a study, involving a Korean cohort, no significant association was found between FCGR2A variants and CD (40), suggesting that the impact of FCGR2A on IBD susceptibility may vary between UC and CD, or between different populations (40, 41). In CD, where transmural inflammation and a more widespread involvement of the gastrointestinal tract are characteristic, the role of FCGR2A may be different due to the distinct immunopathological processes involved. The genetic architecture of CD is likely to involve a different set of susceptibility genes and pathways compared to UC, and FCGR2A may be one of the genes that contribute to the overlap in susceptibility between the two diseases. SERPINB9, also known as Proteinase Inhibitor 9, is a member of the serine protease inhibitor superfamily. This protein primarily inhibits Granzyme B, a serine protease released by cytotoxic T lymphocytes and NK cells. SERPINB9 serves as a critical regulator of immune cell apoptosis and plays a significant role in the functionality of cytotoxic T lymphocytes and NK cells, influencing both tumour immunity and responses to immunotherapy (42, 43). Additionally, SERPINB9 is involved in modulating inflammatory responses by influencing cytokine production and the inflammatory cascade (44). Although direct studies linking SERPINB9 to CD are limited, the protein’s role in immune regulation suggests a potential involvement in the pathophysiology of CD. CD is characterized by an exaggerated immune response leading to chronic inflammation in the gut. SERPINB9 may influence this process by affecting the activity and survival of immune cells involved in the inflammatory response. The diagnostic potential of these genes was supported by validation in an independent tissue cohort.

Our validation at mRNA and protein levels in terminal ileum biopsies confirmed significant upregulation of both FCGR2A and SERPINB9 in CD. While the initial IHC/qPCR cohort was limited (n=6/group), the consistent trend was reinforced by Western blot analysis on an expanded sample set (n=12/group). Nonetheless, we acknowledge that the sample size remains a limitation for broad generalizability, and our conclusions are therefore framed as preliminary, requiring confirmation in larger, multi-center cohorts. The analysis of their diagnostic characteristics revealed that FCGR2A and SERPINB9 have promising PPVs for CD, with 86.8% and 86.6%, respectively. These high PPV values suggest that when either of these genes is highly expressed, the probability of correctly diagnosing CD is high. However, their NPVs are relatively low (30.4% for FCGR2A and 19.1% for SERPINB9), indicating that low expression levels alone are insufficient to reliably rule out CD. This pattern, along with subsequent cell proportion adjustment analysis, informs a more nuanced interpretation of their roles. FCGR2A, whose expression lost statistical significance after correcting for immune cell composition (p=0.411) and showed a very strong correlation with neutrophil infiltration, is best characterized as a sensitive surrogate marker of inflammatory activity, akin to clinically established markers like fecal calprotectin. Beyond our dataset, independent evidence supports this functional interpretation. Integrative bioinformatics and deep learning analyses have identified FCGR2A as one of six hub genes with high diagnostic potential for CD (45). Mendelian randomization studies have further established a causal association between FCGR2A and IBD, proposing it as a potential therapeutic target (37, 46). Mechanistically, FCGR2A signaling drives NLRP3- and ROS-dependent IL-1β production and neutrophil-recruiting chemokines, with responses modulated by FCGR2A genotype (36). Additionally, neutrophil-derived myeloperoxidase has been validated clinically to predict aggressive IBD disease course, reinforcing the relevance of neutrophil-associated markers in disease stratification (47). These independent lines of evidence support that FCGR2A, despite lacking CD specificity, captures a biologically relevant inflammatory signal. Its clinical value should thus be evaluated within a multi-marker framework rather than as a standalone diagnostic test.

SERPINB9, which remained significantly upregulated post-adjustment (p=0.033) and correlated with M1 macrophages, appears to represent a more disease-associated dysregulation marker. This distinction clarifies that FCGR2A primarily reflects the degree of specific immune cell infiltration, while SERPINB9 may signal more intrinsic disease-related processes. Their integration in a diagnostic model is valuable precisely because it captures both the inflammatory context and a more specific disease signal. The high sensitivity (88.65%) but low specificity (26.92%) of FCGR2A suggests that it is a strong marker for identifying CD patients but may also yield false positives. Conversely, SERPINB9 demonstrates a more balanced specificity (46.15%) but lower sensitivity (63.83%), making it a potential complementary biomarker rather than a standalone diagnostic tool. These results highlight the importance of integrating these biomarkers into multi-marker panels or machine learning-based models to improve diagnostic accuracy. Future studies should aim to validate these findings in larger, independent cohorts and explore how these biomarkers perform in combination with other molecular and clinical parameters. In addition, we constructed a nomogram-based prediction model incorporating the expression levels of FCGR2A and SERPINB9 to facilitate individualized risk assessment for CD. The model showed a C-index of 0.819, indicating good discriminatory ability. The calibration curve confirmed that the predicted probabilities closely matched the actual incidence rates, supporting the reliability of the model. In addition, DCA and the clinical impact curve highlighted the potential clinical utility of the model. We note, however, that the performance of this tissue-based model relative to established non-invasive tests (e.g., fecal calprotectin) and its utility in differentiating disease states or predicting outcomes remain to be evaluated in dedicated longitudinal studies.

Our GSEA and GSVA analyses revealed significant enrichment in pathways related to immune signaling, particularly the Toll-like receptor and IL-17 signaling pathways, which are critical in mediating inflammatory responses in CD. The strong correlation between these pathways and the expression levels of FCGR2A and SERPINB9 further supports their involvement in the disease’s pathophysiology. This knowledge not only enhances our understanding of CD mechanisms but also highlights potential therapeutic targets for intervention. Immune infiltration analysis further reinforced the importance of immune cell dynamics in CD pathogenesis. Specifically, we observed increased infiltration of plasma cells, Tregs, macrophages M1, and neutrophils in CD samples, suggesting their active participation in the chronic inflammatory state characteristic of CD. Neutrophils, in particular, are key effectors in the early stages of inflammation, and their strong correlation with FCGR2A suggests that this gene may mediate neutrophil-driven tissue damage in CD (48, 49). On the other hand, the positive correlation of SERPINB9 with macrophages M1, which are known for their pro-inflammatory phenotype, highlights the gene’s potential involvement in regulating macrophage-mediated immune responses in the intestinal mucosa (50). These findings provide a mechanistic explanation for the elevated expression of these genes in CD and highlight their relevance in disease progression.

Furthermore, CPA and Spearman correlation analysis were performed to evaluate the relationship between immune cell infiltration and gene expression in CD. FCGR2A expression was no longer significant after immune composition adjustment, suggesting that its upregulation is largely driven by increased neutrophil infiltration. Despite this, FCGR2A retains diagnostic relevance, similar to other inflammation-associated CD biomarkers such as fecal calprotectin (S100A8/A9) and myeloperoxidase (MPO). In contrast, SERPINB9 remained significantly upregulated after adjustment, indicating a disease-specific expression pattern beyond general immune infiltration. Its strong correlation with M1 macrophages and inverse association with plasma cells further support its role in the inflammatory microenvironment of CD. The integration of FCGR2A and SERPINB9 in the diagnostic model leverages their complementary features: FCGR2A reflects neutrophilic inflammation, while SERPINB9 represents intrinsic disease-related changes. This combination enhances the robustness and generalizability of the model by capturing both immune-driven and disease-specific expression patterns, providing a more comprehensive and clinically relevant assessment of CD.

This study identifies FCGR2A and SERPINB9 as potential diagnostic biomarkers for CD, but their clinical utility requires validation in larger, independent cohorts. Several limitations should be acknowledged. First, the relatively small sample size of the experimental validation cohort, particularly the initial IHC/qPCR cohort, may have limited the statistical power and precision of the estimates and may increase the risk of sampling variability. Second, the clinical validation was conducted in a limited clinical setting and was based on retrospective tissue samples, which may introduce selection bias and limit the representativeness of the study population. In addition, differences in patient characteristics, disease severity, treatment status, and sample processing across public datasets and validation cohorts may introduce heterogeneity and further affect the generalizability of the findings. Therefore, the observed diagnostic performance should be interpreted as preliminary rather than definitive, and the strength of our conclusions regarding clinical applicability should be appropriately qualified. Future studies should validate the FCGR2A/SERPINB9-based prediction model in independent prospective clinical cohorts with standardized clinical parameters, including CRP, fecal calprotectin, and endoscopic disease activity measures. Such studies are warranted to determine whether incorporating FCGR2A and SERPINB9 provides significant incremental predictive value beyond conventional clinical indicators and to further assess their potential clinical applicability in CD. Functional studies are needed to clarify their roles in CD pathogenesis, while blood-based assays could enable non-invasive detection, provided their specificity is established. Standardizing detection methods and addressing technical limitations, such as batch effects and reliance on microarray data, are critical for broader applicability. Longitudinal studies should further assess their correlation with disease activity, treatment response, and relapse risk. Additional research is essential to confirm their reliability before clinical implementation.

## 5 Conclusion

In conclusion, this study provides a molecular characterization of CD through the integration of multi-omics data, identifying FCGR2A and SERPINB9 as key diagnostic biomarkers. The association of these genes with immune cell infiltration patterns and their strong performance in diagnostic models underscores their clinical relevance. Future studies should focus on validating these findings in larger cohorts and exploring their potential as therapeutic targets to modulate immune responses in CD patients. Additionally, the development of non-invasive diagnostic tools based on these hub genes could significantly enhance early diagnosis and disease monitoring, improving outcomes for CD patients.

## Data Availability

The original contributions presented in the study are included in the article/**Supplementary Material**. Further inquiries can be directed to the corresponding author.
