# Supplementary material for: Identification of Crohn’s disease-associated molecular signatures by integrative bioinformatics analysis and experimental validation
Source: Front Immunol. 2026 Sep 29;17:1955481. doi: 10.3389/fimmu.2026.1955481 (PMC13658584; doi:10.3389/fimmu.2026.1955481)
Supplement: Supplementary file 1 [file SupplementaryFile1.docx]

**Figure S1. Correlation between FCGR2A** **and SERPINB9 expression and immune cell infiltration.**

Scatter plots display the correlation between FCGR2A (a) and SERPINB9 (b) expression levels and the abundance of various immune cell subsets. Each dot represents an individual sample, with the x-axis indicating FCGR2A and SERPINB9 expression and the y-axis showing the estimated proportion of a specific immune cell type. The Pearson correlation coefficient (R) and corresponding p-value are provided in each plot. Shaded areas represent the 95% confidence interval of the linear regression line. Density plots along the x- and y-axes illustrate the distribution of FCGR2A and SERPINB9 expression and immune cell infiltration levels, respectively.

**Figure S2. Gene expression levels of FCGR2A and SERPINB9 before and after cell composition adjustment.**

Violin plots with embedded boxplots showing the expression levels of FCGR2A (left) and SERPINB9 (right) in control (teal) and CD (pink) groups, before (top row) and after (bottom row) cell proportion adjustment. The central box represents the interquartile range, with the horizontal line indicating the median expression level. Statistical significance is indicated as *P < 0.05, **P < 0.01, **P < 0.001.
